# Comparative analysis of the *ATRX *promoter and 5' regulatory region reveals conserved regulatory elements which are linked to roles in neurodevelopment, alpha-globin regulation and testicular function

**DOI:** 10.1186/1756-0500-4-200

**Published:** 2011-06-15

**Authors:** Paisu Tang, Stephen Frankenberg, Anthony Argentaro, Jennifer M Graves, Mary Familari

**Affiliations:** 1 Department of Zoology, University of Melbourne, Victoria 3010, Australia; 2 Prince Henry's Institute of Medical Research, PO Box 5152, Clayton, Victoria 3168, Australia; 3 Research School of Biological Sciences, the Australian National University, Canberra, ACT, 2601, Australia

## Abstract

**Background:**

ATRX is a tightly-regulated multifunctional protein with crucial roles in mammalian development. Mutations in the *ATRX *gene cause ATR-X syndrome, an X-linked recessive developmental disorder resulting in severe mental retardation and mild alpha-thalassemia with facial, skeletal and genital abnormalities. Although ubiquitously expressed the clinical features of the syndrome indicate that ATRX is not likely to be a global regulator of gene expression but involved in regulating specific target genes. The regulation of *ATRX *expression is not well understood and this is reflected by the current lack of identified upstream regulators. The availability of genomic data from a range of species and the very highly conserved 5' regulatory regions of the *ATRX *gene has allowed us to investigate putative transcription factor binding sites (TFBSs) in evolutionarily conserved regions of the mammalian *ATRX *promoter.

**Results:**

We identified 12 highly conserved TFBSs of key gene regulators involved in biologically relevant processes such as neural and testis development and alpha-globin regulation.

**Conclusions:**

Our results reveal potentially important regulatory elements in the *ATRX *gene which may lead to the identification of upstream regulators of *ATRX *and aid in the understanding of the molecular mechanisms that underlie ATR-X syndrome.

## Background

ATR*- *X (alpha thalassemia, mental retardation, X-linked) syndrome is an X-linked recessive developmental disorder affecting males. Clinical features include severe mental retardation, mild alpha*-*thalassemia, microcephaly, short stature, and facial, skeletal and genital abnormalities [1-3]. The ATRX protein is large (280 kDa) and contains two highly conserved domains, a PHD-like finger which interacts with chromatin, and a SWI//SNF-like ATPase domain which displays nucleosome remodeling activity, implying that ATRX functions as a chromatin remodeling protein [4-6].

While ATRX is widely expressed throughout development, studies in mice have revealed that ATRX has specific tissue/cell type functions [7-9]. For example, while loss of *ATRX *in chondrocytes has minimal effects on bone growth, mice lacking *ATRX *in the forebrain show apoptosis of cortical neurons leading to a reduction in forebrain size [9,10]. Moreover targeted overexpression of *Atrx *gives rise to phenotypic features that are common to ATR-X syndrome such as severe neural defects and facial dysmorphology [11].Thus, ATRX expression and function must rely on temporal-spatial regulators and/or cofactors for tissue-specific gene regulation and function. While a number of cofactors have been discovered for ATRX including Heterochromatin protein 1 (HP1) [12], Enhancer of zeste (EZH2) [13], Death domain-associated protein (DAXX) [6], methyl-CpG binding protein (MECP2) [14] and Cohesin [15], the upstream regulators of *ATRX *transcription remain unknown and the boundaries of the *ATRX *promoter remain undefined.

In an earlier study focused on the description of the *ATRX *gene and protein sequence, the putative promoter region of human *ATRX *was reported to contain *'multiple CCAAT boxes and binding sites for the CTF family of transcription factors' *[16]. Thereafter, studies have established that the CCAAT motif is not only recognized by the Nuclear Factor 1/CCAAT transcription factor (NF1/CTF) family but also several other transcription factors/complexes which include; NF-E3, GATA1, NF-Y and C/EBP [17]. Thus, relatively little is known about the *ATRX *promoter and the upstream regulatory regions of the gene. The aim of this study was to establish a starting point for experimental studies on the human ATRX promoter by: (i) identifying evolutionarily conserved regions (ECRs) in the mammalian *ATRX *promoter and 5' regulatory regions using phylogenetic footprinting and (ii) identifying putative transcription factor binding sites (TFBSs).

## Results

### Conservation analysis of the *ATRX *promoter and 5' upstream sequence

**Figure 1:**
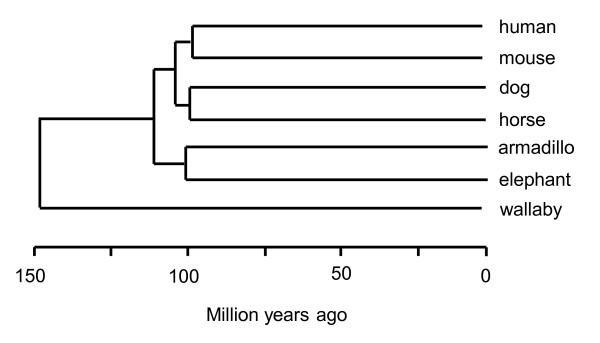
**Phylogenetic relationship between studied species based on fossil and molecular data with branch lengths indicating approximate time since divergence**[64].

The degree of conservation between the regulatory regions of *ATRX *from different mammals, compared with human, were examined across a region spanning -13 kbp to +300 bp, which includes the 215 bp 5'UTR of human *ATRX *sequence [16] since some regulatory elements can lie within the 5'UTR of a gene [17,18]. The molecular timescale of divergence between these species are indicated in Figure 1.

**Figure 2:**
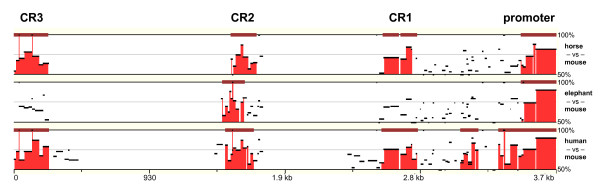
**ECRs in the 5' regulatory region of mammalian *ATRX *genes**. Comparative analysis of the mammalian *ATRX *5' genomic and 5' UTR sequences of representatives of three major eutherian clades - Euarchontoglires (human), Laurasiatheria (horse) and Afrotheria (elephant) - relative to that of mouse (Euarchontoglires), revealed four regions of high sequence conservation represented as standard stacked-pairwise graphs (default settings) on Mulan http://mulan.dcode.org/. Percentage values indicate sequence identity and red shading signifies regions of identity above 70%, calculated over 100 bp.

**Figure 3:**
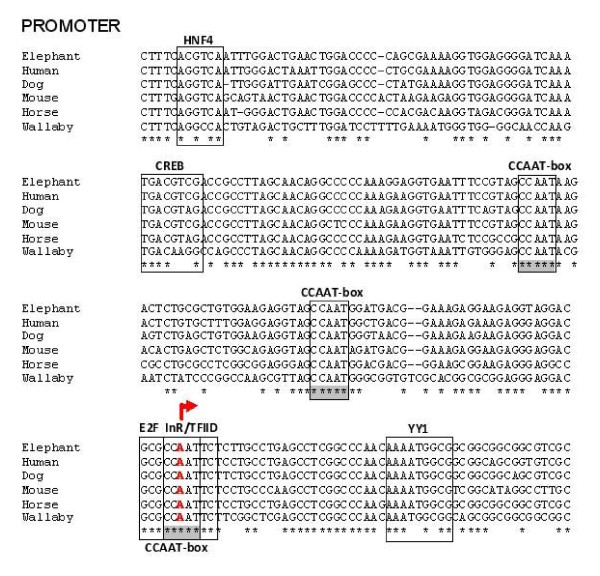
**Highly conserved sequence and TFBSs in the 5' UTR and putative core regulatory region of mammalian *ATRX***. (a) Mammalian alignment showing highly conserved sequence around the TSS (+1 indicated by A) of the *ATRX *promoter. Putative TFBSs are indicated by boxes. Fully conserved bases are indicated by (*) and CCAAT boxes are indicated by (**).

Conservation analysis revealed a clear loss of homology beyond (-500) between human and the most distantly related elephant and tammar wallaby. In addition, three additional dispersed regions of homology were identified by pair-wise alignment of human, mouse, dog, horse, elephant and armadillo regulatory regions. These are located at (-1069 to -693), (-1623 to -1457) and (-12759 to -12669) of the human sequence, and were labelled conserved region 1 (CR1), CR2 and CR3 where CR3 lies most distal to the Transcription Start Site (TSS). Thus, four dispersed regions of high conservation were identified in the upstream regulatory region of the mammalian *ATRX *promoter. These are shown in Figure 2 in a comparison of four distantly-related species representing a broad range of eutherian divergence. Armadillo, representing Xenarthra was not included in Figure 2 and Figure 3 due to the sequence being incomplete. The conserved regions were then examined computationally for conserved, putative transcription factor binding sites.

### Putative TFBSs within evolutionarily conserved regions

**Figure 4:**
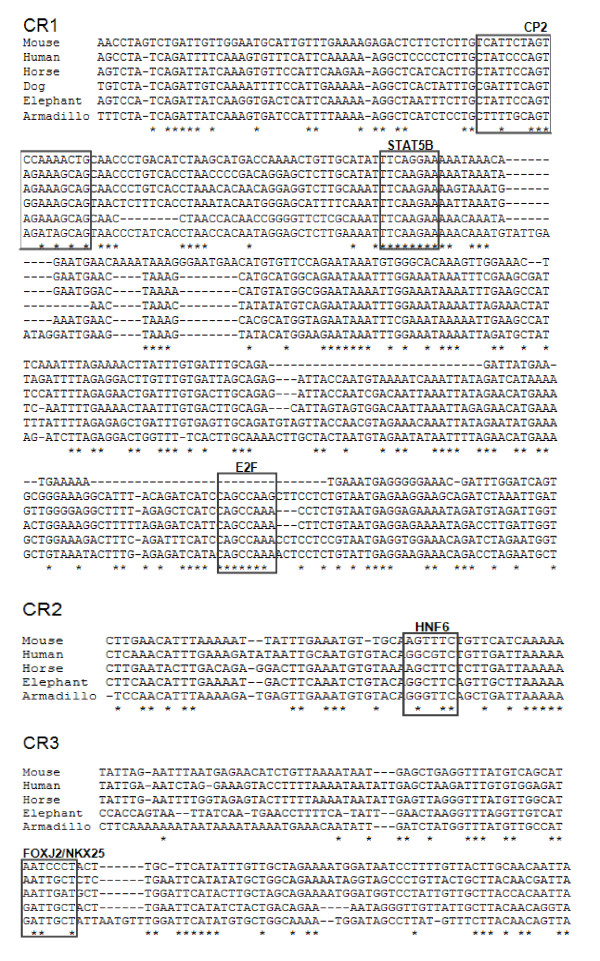
**Highly conserved sequence and TFBSs in the 5' proximal and distal regulatory regions of mammalian *ATRX***. Fully conserved bases are indicated by (*). The coordinates for Conserved Regions 1-3 (CR1-3) corresponding to the human sequence are (-1069 to -693) (-1623 to -1457) and (-12759 to -12669) respectively; however, only the regions containing putative TFBSs are indicated in this figure.

Putative TFBSs within the putative promoter of *ATRX *(-500 to +300) were investigated using the online program, Mulan http://mulan.dcode.org/. Since bioinformatic analyses of DNA sequences typically produce a large number of potential TFBSs and because there is such high sequence conservation in this region, only TFBSs with 85%-100% sequence conservation and consensus identity, as defined by the program matrices, were considered for discussion. These are indicated in Figures 3 and 4.

In addition, a manual examination of the core promoter region for *ATRX *revealed a putative recognition site for TFIID known as the initiator (Inr) element (YYANWYY)[18,19] that is not represented in the TRANSFAC database, and which is fully conserved from human to wallaby (Figure 3). Within the core promoter region, there are also two fully conserved CCAAT-box domains as well as binding sites for HNF4 and CREB.

The putative TFBSs within CR1-3 were analysed by processing different paired combinations of mouse, human, horse, dog, elephant and armadillo sequences with Mulan http://mulan.dcode.org/. This is due to the fact that Mulan requires a relatively high conservation of sequence in order to identify TFBSs. Wallaby was not included in this figure due to low conservation of sequence in this region. The TFBSs found in CR1-3 are indicated in Figure 4 and Table 1 contains a summary of all highly conserved TFBSs in the mammalian *ATRX *5'regulatory regions, in addition to the biological role of the corresponding transcription factors (TFs).

**Table 1:** A summary of the 5' regulatory elements and corresponding TFs found in the *ATRX *upstream regulatory region and 5'UTR

	DNA binding consensus sequence	Putative binding site found in putative ATRX promoter	Biological roles	References
**5'UTR of ATRX (+215 to +1)**				

**YY1** **(+38 to +29)**	SNCCATNTT	CGCCATTTT	Activator/repressor involved in neural development with roles in the testis and meiosis	[35,36]

**ATRX promoter region**				

**Inr element** **(-2 to +3)**	CTTNACC	CTTAACC	Binds to basal transcription factor TFIID	[19]

**CCAAT boxes** **(-2 to +3)** **(-38 to -34)** **(-69 to -65)**	CCAAT	CCAAT	Binds NF-Y, C/EBP, GATA1, NF-E3	[42]

**E2F** **(-6 to -1)**	TTTGGCGC	ATTGGCGC	Family of TFs that regulate mitosis, apoptosis, DNA repair, development and differentiation	[52]

**CREB** **(-122 to -117)**	TGACGTCA	CGACGTCA	Mediation of basal and PKA-inducible transcription	[65]

**HNF4** **(-173 to -168)**	TGACCT	TGACCT	Liver factor involved in liver development and digestive function	[66]

**CR1**				

**STAT5B** **(-963 to -956)**	TTCN_3_GAA	TTCAAGAA	Involved in immune response, erythropoesis and mammary development	[67]

**CP2** **(-1017 to -1003)**	A/TCTGG- CNRGN_6_CNRS	CTATT- CCAGN_6_GCAG	Regulates transcription of *SRY*. Initially shown to bind murine alpha-globin gene	[50]

**E2F (-796 to -791)**	TTTGGCGC	TTTGGCTG	See E2F above	[52]

**CR2**				

**HNF6** **(-1413 to -1408)**	GAATCA	GAAGCY	Liver factor involved in regulation of HNF4 and pancreatic precursor cells	[68]

**CR3**				

**FOXJ2/NKX2-5** **(-12720 to -12714)**	TNAAGTG	TTAAGTA	Involved in regulation of cardiogenesis	[69]

The first intron of some eukaryotic genes, including human, has been shown to contain regulatory elements which influence gene expression [20]. An attempt to align intron 1 of human, mouse, horse and dog *ATRX *revealed little sequence conservation. Pair-wise alignment of these species using both Mulan and ClustalW2 was only achievable for the combinations of human/mouse and human/horse and these alignments revealed only small regions of conservation that did not overlap indicating that it is unlikely that conserved TFBSs are located within intron 1.

## Discussion

A key obstacle in the study of ATRX function has been the absence of identified upstream regulators. The availability of genome data from a wide range of species and the recent development of powerful bioinformatic tools has provided a means of studying the mammalian *ATRX *promoter and regulatory regions. In this study we attempted to derive information on the evolution and conservation of the *ATRX *5'regulatory region for the purpose of identifying putative regulators of *ATRX*, and generate a foundation upon which to design future experiments. In our approach, we make the assumption that the transcriptional regulation of *ATRX *is conserved.

Analysis of the *ATRX *gene revealed high conservation of synteny from humans to birds (data not shown) and high sequence conservation between eutherian mammals and a marsupial, the tammar wallaby. Out of the (-13 kbp to +300 bp) studied, the region corresponding to (-500 to +300) of the human *ATRX *promoter region was found to be the most conserved with 82-90% identity between humans and other mammals studied. The high conservation between human and mouse coding sequences (88%), in addition to high conservation between the human and murine ATRX proteins [21], further supports the extrapolation of data generated from studies in mouse.

The core promoter is the fundamental part of the eukaryotic promoter that provides a platform for the assembly of the RNA polymerase II initiation complex (reviewed in [22]). This region is usually within 80-100 bp surrounding the TSS and is typically a focused region, as opposed to dispersed, containing elements such as a TATA box, BRE, Inr, MTE, DPE and DCE. However, these elements are not universal and not all are present in the same promoters. Moreover, some core promoters do not contain any of the known core promoter elements. Bioinformatic analysis of the very highly conserved sequence around the TSS, which likely comprises the core promoter of *ATRX*, did not reveal any typical core promoter elements; however, manual examination of the core promoter region for *ATRX *revealed a potential initiator (Inr) element (YYANWYY) [19] which is a recognition site for TFIID, a key basal transcription factor (reviewed by [23]).

Analysis of the 5' regulatory region of mammalian *ATRX *revealed several highly conserved, potential regulatory motifs. Only those sites that could be verified in the literature in terms of sequence identity to an experimentally proven consensus binding sequence are summarised in Table 1.

In ATR-X syndrome, mental retardation manifests in approximately 95% of patients and alpha-thalassaemia, one of the defining symptoms of the syndrome, is present in 90% of cases while 80% of patients have genital abnormalities [1]. Significantly, the TFBSs identified in our study correlate to TFs with overlapping roles in neural, globin and testicular development, as well as roles in tissues that are affected to a lesser extent in ATR-X syndrome, suggesting that our findings are not likely to be the result of chance-occurrence of motifs in a DNA sequence. For example, both YY1 and CP2 are involved in brain development [24,25], NF-Y and CP2 have a role in globin regulation [17,26], YY1, NF-Y and CP2 are implicated in the development and function of the testis [27-29], and FOXJ2/NKX2-5 is one of the master transcriptional regulators of cardiogenesis [30] where cardiac defects are observed in 18% of *ATRX *patients.

Furthermore, like ATRX, most of these TFs are crucial for development (YY1, NF-Y, CP2, HNF4, FOXJ2) and ubiquitously expressed (YY1, NF-Y, CP2, E2F), indicating spatial and functional compatibility with ATRX expression.

Interestingly, STAT5B, HNF4 and HNF6 play important roles in liver function [34-36] which do not feature in typical clinical descriptions of ATR-X syndrome yet raises the question of whether *ATRX*, which appears to be quite highly expressed in the fetal liver [31], has a role in liver development and/or function which is not clinically obvious in ATR-X syndrome. Alternatively, HNF4 expression is restricted to liver, kidney and intestines [32] and a regulatory pathway involving HNF4 and ATRX is possible given that renal-urinary abnormalities manifest in approximately 14% of ATR-X patients and gastro-intestinal problems arising from gut dismotility occur in 75% of clinical cases [1]. Furthermore, HNF4 binding sites in many genes expressed in the liver have been found in the vicinity of other liver-enriched TFs such as NF-Y and CREB [33] which is the case in the 5' regulatory region of *ATRX*. Thus, our investigation has revealed a handful of potential upstream regulators for *ATRX *expression which are of biological and clinical relevance. Some of these are discussed further.

The transcription factor Yin Yang (YY1) is a fully conserved, multifunctional protein that can function both as an activator or repressor of transcription (for a review, see [34]). Human YY1 is a sequence-specific DNA binding protein with consensus binding sequence: SNCCATNTT [35,36]. It was later reported that a similar consensus; CCATCTT with flanking nucleotides of G or C constitutes an 'activator' motif for YY1 function with the original consensus being characteristic of a 'repressor' site [37]. The consensus sequence identified in the eutherian alignment corresponds to a YY1 binding site for transcription repression, suggesting that YY1 may be a negative regulator of *ATRX *transcription.

Like *ATRX*, *YY1 *is ubiquitously expressed and genetic ablation of *Atrx *and *Yy1 *in separate mouse studies lead to lethality shortly after implantation, suggesting both proteins play crucial roles in development [6,27]. Furthermore, both ATRX and YY1 have important roles in the development of the brain and nervous system. For example, overexpression of *Atrx *in mouse embryos lead to similar neurological phenotypes of growth retardation, exencephaly and neural tube defects [11] that are also seen in heterozygous mutants of *Yy1 *[24,38], suggesting that ATRX and YY1 could theoretically operate in the same pathway during mammalian neurodevelopment. An obvious hypothesis for the ATRX-YY1 relationship would involve YY1 as a negative upstream regulator of *ATRX *transcription. Such a model may seem spurious in light of the fact that ATR-X syndrome in humans is well documented to arise from reduced levels of functional ATRX protein [1]. However, overexpression of *Atrx *in transgenic mice gave rise to cranio-facial dysmorphology and seizures which are reminiscent of ATR-X syndrome [11], indicating that expression levels of ATRX require strict regulation for the survival of cells and organisms. In addition, deletion of the 5' UTR of *ATRX*, where the putative YY1 binding site is located, has been reported in an *ATR*-X patient [31] and while the precise mechanism of disease arising from this clinical mutation is unclear, we speculate that YY1 could constitute the negative feedback loop for *ATRX *expression while a different transcription factor may be involved in the upregulation of *ATRX*.

Additionally, separate immunostaining of YY1 and ATRX in rodent testis sections reveal a strikingly similar pattern of expression as both proteins are expressed in Sertoli and Leydig cells and in spermatogonia and spermatocytes but not round spermatids and spermatozoa [27,39,40]. Moreover, both YY1 and ATRX have been shown to cause defects in meiosis when ablated in spermatocytes and oocytes respectively [27,41]. Thus, it is possible that YY1 may also regulate *ATRX *in the mammalian testis.

Unrelated promoters for eukaryotic RNA polymerase II have several common elements including the cap signal, TATA box, CCAAT box and GC boxes [42]. We identified three CCAAT motifs at (+3), (-34) and (-65) which are described in one of the earliest publications on the *ATRX *gene and protein [16]. In addition, the spacing between all three CCAAT boxes and between the CCAAT boxes and other regulatory motifs is conserved across all eutherian promoters studied, consistent with findings which suggest that in higher eukaryotic promoters, sequences around the CCAAT box and the spacing between the CCAAT box and other regulatory motifs are highly conserved for specific genes in different species [43,44].

The CCAAT box is estimated to be present in one third of eukaryotic house-keeping and lineage-specific genes [16] and multiple copies are found in promoters of genes regulated during cell proliferation [42]. Notably, ATRX is known to be regulated by phosphorylation in a cell-cycle dependant manner [12] and the *ATRX *promoter with its multiple CCAAT boxes is typical of a gene that is regulated during cell proliferation.

Of all the transcription factors that bind CCAAT boxes, NF-Y (also known as CBF and CP1) can be distinguished from other proteins on the basis of DNA sequence requirements since NF-E3, GATA1 and C/EBP often bind sites containing an incomplete CCAAT motif [42]. High affinity binding sites for NF-Y contain YRR at the 5' flanking end of the CCAAT box and CA at the 3' adjacent sequence. Changes in three or more nucleotides decreases or abolishes NF-Y binding, while one or two nucleotide changes lead to a modest decrease in NF-Y binding [42]. The CCAAT boxes we identify in the *ATRX *promoter contain highly conserved GCG (+2) and TAG (-34, -65) at the 5' flanking sequence and TG (+2), GG (-34) and AA (-65) at the 3' flanking sequence. Thus, by definition, the highly conserved CCAAT boxes at (-34 and -65) would be able to bind NF-Y.

NF-Y is an evolutionarily conserved TF present from yeast to human [45]. A double mutation leads to embryonic lethality in mice as early as 8.5 *dpc *while heterozygous mutations are similar to wild-type [45]. NF-Y has been shown to regulate genes in cooperation with members of the Sp (specificity protein) family of TFs. For example, NF-Y and Sp3 together regulate the *MAP kinase *(mitogen-activated protein kinase) gene [46], and NF-Y and Sp1 have known roles in the regulation of *topo II *(topoisomerase II) [47], *SF*-1 (steroidogenic factor 1) [48] and *SOX9 *(SRY-related HMG box 9) [28]. ATRX, SF-1 and SOX9 play crucial roles in the testicular development pathway and thus it is possible that ATRX, SF-1 and SOX9 are regulated by a similar set of upstream factors - including NF-Y.

The CP2 TF family exists as six isoforms in human (LBP-1a-d, -9 and -32) and four in mouse (CP2a-c and CRTR). CP2, also known as CP2c, has important roles in hematopoeisis, immune response, cell cycle and neural development [25]. These biological processes advocate CP2 to be a temporal, spatial and functionally compatible regulator of *ATRX*. While immune response has not been typically linked to ATRX function, a recent study implicated ATRX and one of its cofactors, DAXX [6], in intrinsic antiviral resistance to the HSV-1 virus [49].

In addition, CP2c was initially identified as an activator of mouse alpha-globin via the consensus sequence CNRGN6CNRS [50] and thus, the putative regulatory pathway between CP2c and ATRX may represent the first direct biochemical connection between ATRX and the regulation of alpha-globin expression. Such a link has been elusive despite the fact that clinical mutations of the *ATRX *gene lead to a downregulation of alpha-globin resulting in alpha-thalassaemia [31]. One possible scenario for alpha-globin regulation is a cascade that involves CP2c activation of ATRX expression which results in ATRX-mediated repression of a negative regulator of alpha-globin expression.

A recent study identified putative CP2 sites in the promoter of testis-determining factor, *SRY*, and showed that CP2 activates transcription of the *SRY *through these sites in a cell-based assay [30]. Binding of CP2 to the *SRY *promoter was confirmed by ChIP analysis in the human testis cell line, NT2/D1 [30]. Within the currently understood casade of genes involved in testicular development, ATRX function lies downstream of SRY and SOX9 ([51], A. Argentaro pers.comm). Thus, in the context of testicular differentiation, CP2 may initiate the pathway for testicular development via activation of *SRY *which eventually leads to ATRX expression. No putative SRY binding sites were identified in our eutherian alignments suggesting SRY is not a direct regulator of *ATRX *transcription.

The E2F family of TFs consists of 8 genes whose products bind to the same consensus sequence: TTTSSCGC (reviewed by [52,53]). Mutational studies of putative E2F binding sites suggest that E2F TFs can function both as activators or repressors of transcription depending upon the promoter context. This is because similar mutations can result in upregulation or downregulation of a gene depending on which gene promoter is being mutated (reviewed by [54]). Interestingly, only a very small number (~23%) of *in vivo *E2F sites actually contain the consensus motif and an analysis of conforming and non-conforming *in vivo *sites suggest that (i) a *bona fide *site must be in the core promoter (+1 to -100) and (ii) the region must be utilised as a promoter in that cell type [55]. One of the two E2F consensus motifs identified in this study (-1 to -6) conforms to these conditions and thus may be a biologically active regulatory element within the *ATRX *promoter.

Members of the E2F family are implicated in a myriad of functions including the regulation of mitosis, apoptosis, DNA repair, differentiation, development and tumourigenesis, with no clear division of labour between members [54]. These roles are very similar to that of the SNF2 family of TFs of which ATRX is a member [16] and recent studies indicate a role for ATRX in apoptosis as inactivation of *Atrx *in mouse forebrain resulted in p53-dependant apoptosis of neuroprogenitors in the developing mouse [56]. In addition, E2F binding sites have been identified in the promoters of many genes intimately involved in the regulation of cell cycle progression [54] and *ATRX *could be one of these genes since *ATRX *null mutations can affect cell-cycle progression and interfere with proper chromosome segregation in both somatic and germ cells [41,57].

## Conclusion

We identified twelve highly conserved TFBSs in the 5' regulatory region and 5' UTR of mammalian *ATRX *that are likely candidates for regulating *ATRX *expression. Future experiments involving DNA binding, mutational analysis, ChIP and transactivation assays will verify whether these sites are *bona fide *regulatory motifs. Furthermore, the identification of ECRs in the *ATRX *promoter region could provide a starting point for experimental studies to define the minimal promoter of *ATRX*. While a literature search did not reveal any idiopathic cases of ATR-X syndrome which are could be attributable to mutations in the 5' regulatory regions, an understanding of how *ATRX *is regulated is an important component of understanding its functions during development and other important biological processes.

## Methods

To compare the human *ATRX *5' regulatory region to that of an evolutionarily divergent range of species, input sequences were retrieved with BLAT searches [58] at the UCSC Genome Browser http://genome.ucsc.edu/ or by BLAST searches of whole genome shotgun databases, using human sequence corresponding to the 5' regulatory region of the *ATRX *gene (-13 kbp to +300 bp). Only species for which an *ATRX *orthologue was definitively identified and for which sufficient 5' sequence was retrievable were included in the generation of each results figure. These belonged to human (*Homo sapiens*), mouse (*Mus musculus*), dog (*Canis familiaris*), horse (*Equus caballus*), elephant (*Loxodonta africana*), armadillo (*Dasypus novemcinctus*) and tammar wallaby (*Macropus eugenii*) (Table 2).

**Table 2:** References to the sequence data used in this study

Species	Reference sequence	Source
**human**	NCBI36/hg18 chrX: 76928075-76941134	UCSC Genome Browser http://genome.ucsc.edu/

**mouse**	NCBI37/mm9 chrX:103124406-103128433	UCSC Genome Browser http://genome.ucsc.edu/

**dog**	Broad/canFam2 chrX:63091021-63104321	UCSC Genome Browser http://genome.ucsc.edu/

**horse**	Broad/equCab2 chrX:57708104-57721404	UCSC Genome Browser http://genome.ucsc.edu/

**elephant**	Broad/loxAfr3 scaffold24:23535243-23535990	UCSC Genome Browser http://genome.ucsc.edu/

**armadillo**	AAGV020428528	GenBank

**wallaby**	ABQO010522248	GenBank

Multiple sequence alignments were compiled using Mulan in 'TBA' mode http://mulan.dcode.org/[59,60]. Candidate TFBSs within ECRs were identified using multiTF [60][61] from the Mulan website, selecting the TRANSFAC professional V10.2 TFBS database for vertebrates, with matrix similarity set at 0.85, 0.95 or 1.0, and using only high-specificity matrices. The analysis was performed on large regions of upstream genomic sequences as well as on individual, short ECRs identified from the initial alignments. Identification of TFBSs was performed on alignments of two-to-several species from diverse taxonomic groups and only those conserved in at least two species are reported here. In addition, Map Viewer http://www.ncbi.nlm.nih.gov/mapview/[62] was used to investigate the conservation of synteny around the *ATRX *gene between species, Readseq http://www-bimas.cit.nih.gov/molbio/readseq/ was used to convert sequences to FASTA format, and ClustalW2 http://www.ebi.ac.uk/Tools/clustalw2/index.html[63] was used as an additional sequence alignment tool.

See Additional file 1: Input sequences, for the original data used to perform our analyses.

## List of Abbreviations

ECR: evolutionarily conserved region; TFBS(s): transcription factor binding site(s); 5'UTR: 5' untranslated region; TSS: transcription start site; CR: conserved region; TF: transcription factor; DNA: deoxyribonucleic acid; bp: base pairs; kb: kilobase pair

## Competing interests

The authors declare that they have no competing interests.

## Authors' contributions

PT conceived of the study, PT and SF designed the study and carried out the experiments, PT, SF, TA, JMG, MF wrote and drafted the manuscript. All authors read and approved the final manuscript.

## Supplementary Material

Additional file 1 **Input sequences**. The input sequences used in this study are provided in FASTA format Click here for file
