# Supplementary material for: Comparative analysis of the ATRX promoter and 5' regulatory region reveals conserved regulatory elements which are linked to roles in neurodevelopment, alpha-globin regulation and testicular function
Source: BMC Res Notes. 2011 Jun 15;4:200. doi: 10.1186/1756-0500-4-200 (PMC3144453; doi:10.1186/1756-0500-4-200)
Supplement: Additional file 1 — Input sequences. The input sequences used in this study are provided in FASTA format [file 1756-0500-4-200-S1.PDF]

Additional File 1: Input sequences used in this study

Figure 2

>mouse

```
tattagaatttaatgagaacatctgttaaaataatgagctgaggtttatgtcagcataatccctacttg
cttcatatttgttgctagaaaatggataatccttttgttacttgcaacaattaaaattaatttcccccca
tcgcctttataatatattctctttataggaaaatttgtgagtaatggaaaagtacagtgcagaaaaaaa
tcactgagacatttcatctgaaggtatcattgaaaaaaaaatgtatgcacagagagagagacagagaca
gaaagagacagacacaaacagagagacagaaaaatagacagatagacagagtacctctgagatctggtc
tctcttttatccctggcttccctggaactcacagagattcacctgcctgtactgtctgagtgtgggga
ttaaagatgtacacaaccatgcctggctatatttatgtttgtgtgtgagtgtgtgtgtgtagttttca
cactgttggagatacacatggagctgaggtaatgggcagctgtgaattgcctgagtaactgttgcaaac
tgaatgcaggttctctgcaagactaatatatactcataaccatggcatcatctctccagcagcgtcagg
gcaggcatacatggtgctggagaaggaactgagatttcgacatcttcatccaaaggcagcagaaggaga
ctgtgagtgacactgggtatagtttgagcataggagacctcaaagcccacctccatagtaacatacttc
ctcttacaaggccacacccactccaacaaggccatgcctcctaatagtgccagtccctatgggctaagt
attcaaacacatgagtctatggggaccatacctattcaagtcacacactgggctatagagtaaaatgat
gtttaaaacaaacagacaaacaaagacccctaacaatacagaatggaggcaaatgccagtaatcccagt
gctcagagtctcagccaggagttcaagaccatccttttcatgctgtgataaaatgttctgacaaaaaca
acttaaggaagaagggtttaattgtctcacagttccaggttatacagttacacaaggtggcaggaactg
gaaacaactggttacatcacatctacagtcaggcgtagagagaaatgaactcatacttgcatgcttaat
gctcagctccatttttttcattcttacgcagttgagagtccacagccttgggaaatgtgttaaccacagt
gggaaggtctccctacctcagttaatacaatcaagataattccctacagtcatccttagaggtccttct
cccaggtcattgtgtcaaagtggtggcaattaaaactaaacttccagctacatagtgagtttgaggcca
gtctgggctacacaaaaccttgtcccaaaaacaaaacaaacaaacaaaattcattcagattaaaataat
agatgaattactatttttttccatagttgtgtctgttagatagcaaggatatttgttgtgatgaaataga
tctttcaggttaacttcatttctcaaggaggaagaacatggcttctgtaaattgtgtttaagtaatatt
taatatattaaacacaaaagaagaacattattgaacaacagaagattgatttccacagttggtagtgga
tcttgaacatttaaaaattatttgaaatgttgcaagtttctgttcatcaaaaatgtatgctatccaaga
gaactctttaaaattgtgatgatgttgacgtatatagctctccatacttgatggcttctgggtgtggga
gatggcatctgggggacaggctcagttttctttaaagggctggccactgggagtttgaccaggctatgg
ataacacaaattggtcttgtttcttctcctcctcctctttctcctcctctttctcctcctccttctcc
ttctcctcctccttctgtttcttcttcttctcctccttctcctcctcctcctcttcctccttcttttcc
tcctcctcctcctcccttccttcttccagggagggcagacttgagaggactaggaagtgagtgcgatgg
ggtttcacaatgtcaaattccaaataatcaatagaagtactgtgaaaagacataaaaaaatgtatatta
gaggctgaagagactgcttagtgattatgggcacttgctgttcccagcacctacattgggtaggtcata
cctgcttgtaactccagtgccaggggtccaacattctcttctgcagtgtgaggacttgtgtacatatat
acatccaggctctctcaaacatacatacaacatttaaaaaccaaatctttatatgttagttagccaggt
gtgatggcatatgcctttagtctaagttctcaggtggcaaaggcagttggatttctgagctgaaagcca
gcctgatctacagagcaagttccaggagagccagggctgcacagagaaactctgtctcggaaacaaaca
aacaaacaaacaaacaaaaacctagtctgattgttggaatgcattgtttgaaaagagactcttctcttg
tcattctagtccaaaactgcaaccctgacatctaagcatgaccaaaactgttgcatatttcaggaaaaa
taaacagaatgaacaaaataaagggaatgaacatgtgttccagaataaatgtgggcacaaagttggaaa
cttcaaatttagaaaacttatttgtgatttgcagagattatgaatgaaaaatgaaatgagggggaaacg
atttggatcagttggtaagtgagatataccccccaatcatatcagattgaactaatggctgacctgtgtg
tgacaatttcaagattgttcaatatcaaaagaattagatgtcaatgtccagttgatcatatatatataa
atacctgtaagtatccttaagtatgatggggtacacctgtcttctaagcactttgggaggctgcaagga
cagtccaagtttgaggccagcctcatatacatgttgagttgcagcctagtcaggactgtatagctagaa
tttttctcattctctttccacaaaaggtaaaaattcacacgtgtaagcgttggcaagctgaatattata
atgaaatatatagaagaaaaattaagaatattataaaataggaaagaaaatatttatatttgggtggc
tatggagattaatgattaagagtgtgcatgtgggggaggggttaggacatagctcaatgatagagtatt
tgcctaacatgcacaaagccttgggtttaaaacagcataagaatgtatgtccaccctccccgcaaggat
ggatgtttggctgaatgtggaacttgtgcattggtatcctcacccattaaaatgttaggtctgcctact
ttggatggttcaagcatgagattaagataaaggatctgtgtagtggtcgacatatagtaaattctcaat
aaacgtaaatggggtgtcattgtttcaccaattatgtggaagtagagaggttggaccaagagcgcattt
tcaggataatgtcaagatacatccctgacactttgaggtcagcagtaactgaactggaccccactaaga
agaggtggaggggatcaaatgacgtcgaccgccttagcaacaggctcccaaagaaggtgaatttccgta
gccaataagacactgagctctggcagaggtagccaatagatgacggaaagaggaagagggaggacgcgc
caattctcctgcccaagcctcggcccaacaaaatggcgtcggcataggccttgctttgtttccgtggct
c
```

>human
tattgaaatctaggaaagtaccttttaaaataatattgagctaagatttgtgtggagataattgctctc
tgaattcatatatgctggcagaaaataggtagccctgttactgcttacaacgattaaaattatttcatt
tctcccatcatctttataatatgttctcgttataggaaatctggaaaaatggaaagtacaatgcaggaa
aaaattactcagccatatcacctgaagatatctttccattgttggatgtgtcctccaggccttttttct
cttataacacatcatctgacttgtttcttcttctgaacatagcatgtgactaccttgactttgataat
ttgaaaaatgaagcgttattgtaataacttagtatatgttaatgacatttatcatttaaatatataatt
ttcttgatgttcccccaaactaaaaaatctgttattttgtcatagatagcttcttaaaattccttatct
tgctgtttcttcttcagtatgtctatgaggttttacttcactctatttgtaattaggatctctgtct
ggttcattttcaaatatatctgatcttttattttcgttttagtaactcttgttgaaagattagataatt
tggagagtaaacaccttaaatataagatttccttaagtttagaatctttaaaaactattgcattattaa
ttgaactttcactggtttatgagcaggaaacatctggtttggggctatcagattaagtttgtttgattt
acttttttttttttttaagatggagtctcgatctgtcacccaggctggagtgcagtggcatgatctcgg
ctcactgcaagctccgcctcccggattcaggccattctcttgcctcagcccccgagtagctgggacta
caggcgcccaccaccacgcccggctaatttttttgtatttttagtagagacagggtttcaccagattggt
cccgatttcctgacctcgtgatccacccgcctcggcctcccaaagtgctgggattacaggcgtgagcca
ccgcgcccagccttgatttactgttaaagattgtggttaggcatggtggctcacacctgtaatcccagt
actttgggaggctgagatgggaggattgcttgagtccaggagtttgagaccagcgtaggcaacacagtg
agacaccttatttatttatttatttatttaagatagagtctcactctgtcgcccaggctggagtg
cagtggtgtgatcttggctcactgcaacctctgcctcccgggttcaagtgattcttctgcctcagcctc
ccgagtagctgagactgcaggtgcccatcaccacgccccactaattttttttgtatttttagtagagat
ggggtttcaccatgttggaaaggctggtctcgaactcctgacctcaagtgatccgcccgccttggcttc
ccaaagtgctgggattacaggcatgagccaccgcacccaacttgccctctctattaaaaaaataaaaaa
taataataataataaaaactggtggggtggtgagtgtctgtagtcccagctacttggcaggctgaggca
ggaggattgcttgaacagaggtcagtaagctgtgcttgcatcactatactccagcctgggcaacagagt
gagactgtctcaaaaaaaattgcatttgtatggttcatttgttataactgatatattattattaactaa
agtccacagtttactaagagttcacagtacataagagtttttgtagtactttctatgggttttgacaaa
tttataatgacatatattcaccattctagtatcaaacagaatagtttcactgcccaaaaattccctgtg
gtccaccaatttatcagtttccctccttgccatgtcccctcaacccctggaaaccactaatcattttac
tgtttccatagtgttggcttttctaaaatgttatatatttggaatcatacaatatgttgcctttttaga
ttgtcctttttttttttcacctcagcctctcaggtggctgggaccacaggcatgcaccaccacacctgga
taattttgtattttttttgtagagacagggtttctccatgttgccctgtgtggtctcgaactcctgggc
tcaagcaatcccttctgcctgagcctcccaaagcgttgggattacaggcgtgagccactgtgctgggac
taggttggcttcctcatcttagcaatatgcatctcaggttcctccacgtcttttcatgacttgatagct
catttattttattgccaaataatattccattgtatggattaccacagtttatccattcatatattgaa
ggacatcttggttggttccaagttttggcagttgttaataaagctactataaacattcacttcaaggtt
ttgtgtagatgtaaaattttcaactcatttaaccaaggaatgcaatagtgggataaatgataagggtag
gtttagcggctgggtgcagtggctcacgcatgtaatcccagcacttgggagaccaaggcaggtggatca
cttgagctcaggagttcaagaccagcctggccaacatggtgaaaacctgtctctactaaagaaaaaaca
aaacaaacaaacaaaaaaaaaaaaaaagagagagagagagagagtacgtttagcattgtaagaactggc
caaacgtcttttcaaagtggctgtactattttgcatttcatgcagcaatgaatgagagctcctgttcct
ctacatccttgtcagcattttgtgttgtcactgttttggattttagccattctaataggtatgcagtgg
tatcatattgttgttttaattcacaattccctaatgacatatgatactgagaagctatttgccatatgt
atatcttctttggtgaggtttccaagttttttgtccatttttaaattaggtctttgttttcctattgtt
gagtgggaagatttcttatattttggataccagttctttatcaaatatgtgttttgcaaagattttctc
tgtctgtggcttgtgttttcattcccttaacaatttcttttgcagagcacaagttttccattttatttt
atttttatttttttgaggacaagtctccctctttcgcccaggttgaagtgcagtggcaccatcttggct
caccacaacctctgcctcccgggttcaagcgattctcctgcctcagcctcccgagtagctgggattaca
ggtacgccctaccacgcccagctaatttttgtatttttagtagagatggggtttcaccattttggcag
gctggtctcaaactcccgacctcaagtgatccgctcgtctcggcctcccaaagtgctgagattacaggc
gtgagtcaccgcgcccggccaagtttttcactctgttgcccaggctggagtgcagtggcatgaactcgg
ctcactgcaacttcccccacccgggttcaagtgattctcctgcctcagcctcctgagtagctgggatta
taggcgcccgccaccatgcccagctaattttgtatttttagtagagatggggttttaccatgttggtc
aggctggtctcgaacccctgacctcgtgatccgcctgcctcggcttcccaaagtgctgggattacaggc
gtgagccactgcatctggccccaagttttccattttaataaaatccaacttatcaatttttctcttat
ggatcatactttttgtgttatacctaagaagtcattgtcaaacccaaggtcatctggattttatcctgt
gctatcttctagggattttatagctttgtgttttaaatttagatctatgatccattttgaattaatttt
tgtgaaaagtgtaaggtctatgtttagagtctttttttttttcgtttctttgtcagagatcagttgact
atatttgtgtgggtctatttctggtctctctattctatccctttggtctatttgtctattctttcacca
ataacatttgaattttaaattacattcatgttttgaataggatatacactaagatggtaccaactata
aaaggtaaaaaagaatatatattgaaaagtttccctcctgtcctcttccccacctaccagattatcttc

cttaggcaactactattatcaaatttagttttaattctttcagagttattttgccttttatcttctctg
tctcttatctaacacagatattaaaatactatgcatggtgttacgcaactgcttttttttgttaacaatg
taactaggaaatcatcccatatctgtacatctagacctgcctctttaaaatgttatttttattgtggta
aaaaaatatgtaacattaaatttaccctcttgaacatttaaaaaaatgtctataaatttatggtgtata
agtacaattttgttacatgcatagattgcatagtggtgaagtcagggcttttatagtgtccatcaccca
aataatgtacattataccttttctttgtttttttttttttttttttttttgagatggagtctcgctctgt
tgcccaggctggagtgcagtggcgtgatctcggctcactgcaagctccgcctcctgggttcacgccatt
ctcctgcctcagcctcccgagtagctgggactacaggtgcccgccaccgcgcctggctaatgttttgta
tttttagtagagatggggtttcaccgtggtctcgatctcctgacctcgtgatccacccatctcggcctc
ccaaagtgctgggattacaggtgtgagacaccacacccggccccccgccctttttttttttgagacatg
gtcttgctctgttgaccatgttggagtgcagtggcgccatctcagctcattgcaacctccgcttcctgg
gttcaagcaattctttcacctcagcctcctgagtgctgggactacaggtgtgtgccaccacacctggct
aatttttgtatttttttggtacagacagggtttcgccatgttggccaggctggtctcgtacttctggcct
caaatgatcctcccacctcagcctcccaaaatgctgggattgcaggcatgagccaccaagcctggtgaa
aagctttttttttgtttttttattttttttgagacagggtctcactctatcacccaggctacagtgcagt
ggtgcaatcttagctcactgcaacctctgtctcccaagttcaagagattctccagcctcagcctcccga
ctagctgggactagagatgtttgccaacacctgtttccctgcctattttttgtattttttaatagagatgg
ggtttcaccatgttgtgcaggctcttctcgaacttctggcttcaagtgattcgcctgcctgggtctccc
aaagtgctgggattacaggtgtgagtcaccatgcctggcctcattatacctattttttaagtatacagct
tagtgctcttaagtatactcatgttgttgcacaacagatctgtagaatcttttcgtgttgcaaaactga
aactctgtatccactaaacactaaatcctctccgtcaacctttgttagccacctttctactttttgttt
ctatgtttcgactactttagatacttccagtggaatcatacactatttatcctttttgtgactggtttat
cttccttaacatagtatctttaaggtatcatccatgttaaaaaaaaaaaaggatttccttcttttttttaa
ggctgcatacaattccaatgtatgtatacaccacgttttctttatccattcatctgtcaatggacattt
ggattgaatctacctcttggctatggtaaataatgggatagtgggtcaggcgtggtggatcacgcctgt
aatcccagcactttgggaggctgaggcaggctgatcacttgaggtcaggagttcgagaccagtctggcc
aacatggcaaaaccccatctctactaaaaaatacaaaaattagctgggtgtggtggtgcatgcctgtaa
tctcagctactcgggaggctgagacaggagaattgcttgaacctgggggggcagatgttgcagtgagctg
agatcatgccactgcactccatcctgggtgacagagtgagattctgtctcaaaataaataaataaataa
taatgctacagtgaacatgtgtgtgcagatatctttctaatatacaaatttgaattgttttggatatac
acccagaaataggattgctgaatcacatggtaattccatttttaactttttaaagaatctccacacttt
tttccataatggctggaccattttacattcccgctaacagtgcacatggattctactttctcctccatt
ttgctgacatttactattttctgttcttttgttagcatacatcctaaagggtgagcagtgatatctgat
tgtgtttttgtttgtttgtttattttttgagacggagtttcggagtttcgctctgtcgtctaggctggag
tgcagtggcgcgatctcggctcactgcaagctccgcctcccccgggttcacaccattctcctgcctctc
cagagtagctcggactacaggcacccgccactacgcctggctaatttttttgtatttttagtagagatgg
ggtttcaccatgttagccaggatggtctccatctcctgacctcgctatccgcccatcttggcctcccaa
agtgctgggattacaggcgggagccacggcgcccggcctgattgtggttttaatttgcatttcttttat
gattagtgatgttgagcatcttttcatatgcttaatggccaatttatatttctttagagaattgtcca
ttcaagtcctttgcacattttttgaatttttttttagaagtcccttttatattctgaatattaacccatta
tcagatatacgacttacaaatatttctctcatttcataggttgtcttttcactcggttgatagtttct
ttttacgtgaataagtttttaagtttgatgtagcctcacttgcctttttttgcttttgttgcttgtgct
tttggtgtcatagccaagaaataattgccaaatataatgtcctgaagcttttcgtctatgttttcttcc
aggagttttatagttttatgtcttatgtttagttttttaatctgctttgagttaatttttgtatgtggt
gtcagataagggtccaacttcattcttttacatgtggatatctagtacttctaacaccatttattgaag
aaactgtcctttctcattgtgtagttttggaatccttgtcaaaaatcatttggccccctgggcaggtgca
gtggctcatgcctgtaatcccagcactttgagagactgaggtgggttgatcacatgagctcaggagttc
gagaccagcctaggcaacatggtgaaaccccacctctacaaaaaatgcaatgattagccgggcatggtg
gcacatacctgtggtcccagctacttgagaggctgaggtgggaggatcacttgagcccaggaggaggag
gctgcagtgagccatgactgtgccactgcactccagcctgggtgatagacagggagagagaccttgtcg
caaaaaaaaaaaaaaaaaaaaaaaaagaagaagaagaagaaaattatttggccatatatgcaagggtttat
tattctgtttcattggtccatatatctattttttaagtcagtaccacaccatcttgtttactaatcagga
aatttgtggcccccagctttgctgtttttttttgtttgtttttgttttttttttttttttgagattgtttt
agcttttcagcatcctttgagatcccacggaaattttaggatgggtttttctctgcaaagaatgccatt
gggattttgatagggattgcattgaatctgtagattcctttgagtagtatggacatttttacaacatta
agtcctctaattcatcagcatgggatacccttctatttatttgtatcttcttttatttcttttaggtat
gttttggagtttttagtgtgtaagacttttacctccttggttaagtttattctaaacatttttatcctat
ttgatactatagtaaatgggattgttttgttttcttaatttcctttttttttttcttttttttttagacagg
gtcttactctattgcccaggctggagtgcagtgatacaatcttggctcactgcaacctctgcctcccag
gttcaagcaattctcctgcttcagcctcccaagtagctgggactgcaagcacatgccaccacgcccggc
taatttttgtatttttagtagagatggggtttcaccatgatggccaggctgctcttgaactcctgacct
catgtgatccacctgcctcggcctcccaaagtgctgggattacaggcgtgagccaccatgcccggcctt
aatttcctttttggattggtcattgttagtacatagaaatgcaactgacttttcaaaaaaacttatgga

gctatatggtatttcctgtgtggatgtaacataatttatttaaccagtcttctgttaaggagcatctgg
gttgtttctaatatatatatatatatatatatatatatatatatatatatatatatatatatatatttata
gagacagtgtcttactatgtttcatatatatatatatatatatacacatatatatgtatatatttatag
agactgtgtcttactgtgttacccaggatggtcttgacctactggggtcaagcaatcctcccacctcag
cctcccaaagtgctgggaatacaggggtgagccgccatggctggctggctttttttgtttgtttgtttg
tttttcagagatagggtctcactatgttgctcaggatggccttgaacttctggcttaagcaatcctc
ctacctcagcctcctgagtagttggaactacaggcccacaccactgtgcccagctatccagtgttttga
tgttacaaatgatgccgcagtgaataatcttgtgtacatctcatttgtccctgtaaagtatatctgtt
ggataaattcctagaagttaaattgctgggttaaatatatatgtattttaaaatttcataaatattgc
tcaagaattatgattctgacataaaataggggtttgaagctgtttgtttgcttatttatttatttatt
ttcagatggagtctcactctgttgcccaggctggagtgcagtggcataatctcaagtcactgcaacctc
tgtctcctgggctcaagcgatgctccctcctcagcctcccaagtagctgggattacaggcacacaccac
tacgcccagctaattttttttgtgtgtgtatttttggtagagacaggctttcaccatgttggccaggctg
atcttgaactcctgagctcaagtgatccactcactttggcctcccaaagtgctgggattacaggcttga
gccactgagcccagccaaagctgtatattaaaaattatggagtaactattttctcagctgatttgctct
ctttcaggtactgtatctcttattccttccaaaacaaataagcacattcacaagttacgcatttatggt
caagagtcagagaaaggtctaggttgccttctgtggcaatttgaagaaaaatgtgaagatgtgtaccag
taaacttcttctaaaatagttgatgggaacccgttgataatcatcactgaaagcgatacacctatatgt
atatcagtaaaactttgtcggccatcctttactaacaaaggaattctttttctttctttctttttttt
tgagatggagagatggagtcttgctcttgttgcccaggctggagtgcagtggtgcaatctcggttcact
gcaacctccgcctcctgggttcaaccaattctcctgcctcagcctccccagtagctgggattacaggca
cccaccaccacacccggctaatttttttatatttttagtagagatggggtttcaccatgttggccaggct
ggtcttgaactcctgacctcaggtgatccacctgcctcggcccacccaaagtgctgggattacaggcatg
agccaccgcgcctggcctctttttctttttttttttaagtaaaaaaaaaatttttttttagagagggagcc
tcactattttcccagtttggtcttgaactactgaatttaacccatcttcccttcttggccttccaaag
ttaagagattacaggcatgagccaccatgttcggctcaggtattctcttatgcttcacaaatatagtga
gttgttattcacaatgtattcacaagttgttattcacaacaatattcacaaatgttgttcattttgaaa
atgctgtgattattgctgggcatggtggctcacgcctgtaattcaagcactttgggaggttatggtggg
aggatagtttgagctctggactttgagacatgagcctgggcaacatcgagacctactctctaaaaaaat
aaaacaaaaagataaaattagccgggctgtggtggcttaggcctgcagttgtagctacttgggaggctg
aggtgggaggatcacttgagcttgggaggtcgaggctgcagtgacccatgatcacgccattatgctcca
gcctgggaggccaagtgaggccctgtctcaaaacaaaattaaaatttaaaaaatctatgattgcttggc
atggtggctcatggctgtaatcccagcacttttgcaggccgaggcgggtggattacctgaggtcaggag
ttcaagaccagcttggctaacatggtgaaaccccgtctctactaaaaatatgaaaattagttagtcgtg
gtggtgggtgcctgtaatcccagctactagggaggctgaggcaggaaaatcgcttgaacctgggaggta
gtggttgcagtgagccgagatcatgccactgcgctccagcctgcagcctgggctacaagagcgagactc
catctcaaaaacaaaacaaaacaaaacaaaacaaaaacaaattgtgattatagttttattattaagttt
cttttgtgagacttgtcatttaaagctgtttagtgttgagaagtctctcagcaacagctagtttctacc
ttaataatttcaattcatttaaaaataatgtgaggcaggagaatagggtttggaggcagggaacctaag
gctgagtaaagttgacttcctagaactgaatcaaaaggaaaaccccaactttccacacctaagtaacaa
aaagactactccctttgcaaatcctcttccttttccgcttggcagatggaaaactgaaagtatctctga
ttggttgcagaaagcagatgtttgtatagcagtgtaactttgtaacttcactttagcctctgattggtt
gctgtccacaaccaattagatgcttgcatagggtgtgacccttgtaacttcactttagcctctgattgc
gggccactactttatttacatagggtgtacaccaagtaaccaatggaaaacctctagagagtatttaaa
tcccagaaaattctgtaatgggggtcttgagcccctgtgctctggcagctcctgcagtcttgtggagtg
tactttcattttcaacgtgtctctgcttttgttgcttcattctttccttgctttgtgcatttgtccaa
ttccttgttcgaaacgccaataacctggtaacatgttgactatttactatttttctcatagtgaagtca
gttgaacagcaagcacatttctgtgctgccttgtgatggagtacatcattcaaattcactgcatttacc
aaggagaaaaaaatgactcctgtaaattgtatgttgaaatgatatttaatgtgtacaagacaaaatcag
tatagcaggtacagaggtctggtttctataattgccaactgaactcaaacatttgaaagatataattgc
aatgtgtacaggcgtctgttgattaaaaagttgcctgaggccaggcgcggtggctcacccctgtaactc
cagcactttgggaggtcgaggttggaggatggcttgagcttagatgtttgagaccagcctgggcaacag
acagacctacctcatctataccaatacataaatacataattagctgggcttgatggcacgcgcctgcag
tcccagctgcttgagaggctgaggtgggaggattgcttgagcctaggaggtcgaggctgcagtgaactg
cgtcacgccactgcactccagcctgggtgacagagctagaccctgtctcaggaaaaaaaaaaaaaaaaa
gttgcctgagcctatcagattttcaaagtgtttcattcaaaaaaggctcccctcttgctatcccagtag
aaagcagcaaccctgtcacctaaccccgacaggagctcttgcatatttcaagaaaaataaatagaatga
actaaagcatgcatggcagaataaatttggaaataaatttcgaagcgattagattttagaggacttgtt
tgtgattagcagagattaccaatgtaaaatcaaattatagatcataaaagcgggaaaggcatttacaga
tcatccagccaagcttcctctgtaatgagaaggaagcagatctaaattgattggtttgtgagctaaaca
tcacatcaaattggggaaatggcccgtgtgtgtgtgtgtgtgtgtgtgtgtgtgtgtgtgtattctaag
agacactttattcaatatggaaagaattttatgcagcgttcaagttggtcttatgtcgtctgcacacat
atacatatatataagtatattatacacatatacatttccaagcatgttggtgtgccaaatattatgtat

aaaacatctaggaaagcaaataacttgatgatagcctagcatataggaaagaaaatacttacataggat
ggccatacagattagtggttaagagtgtctggtgaatctcggctccactactgtgggactttgtgtctt
ggtttcctcatctggaaaatgtaggtttgtctacctcagagagtgtaaagattaaatgagataacccgg
ataaaggattagtgtagtgccgagcatatagtaaactctcaataaatgtaggttgctgttattacttcg
ttaatatgtcaaagaaaaaagaggttggaccaaaagagcattttcaaaataaagtcaaactatgtcact
agcactttcaggtcaattgggactaaattggaccccctgcgaaaaggtggaggggatcaaatgacgtcg
accgccttagcaacaggcccccaaagaaggtgaatttccgtagccaataagactctgtgctttggagga
ggtagccaatggctgacggaaagagaaagagggaggacgcgccaattctcctgcctgagcctcggccca
acaaaatggcggcggcagcggtgtcgctttgtttccgcggctc

>horse
tatttgaattttggtagagtactttttaaaataatattgagttagggtttatgttggcataattgatgc
ttggattcatacttgctagcagaaaatggatggtcctattgttgcttaccacaattaaaattatttaac
tttccctaccagctttatgatatatgcccattgtaggaaatttggagaaaatagacaagtagaaagcca
gaaaaaaaaccacccagacatatcacttgaagaaaactaatctatattttggaggtatcatccaaacc
tttttgctctaaagcatttctttaaaatggaatttaaaaaattactcagtataaaggctaaatttctgg
aactcagggtaatcctttaatgacttgtagcattcccattgtgagtaatcgccgactttgttttcttct
tcagaacatcacacatgacaaccttgattttggtaatttgaaaaataaggcattactacaataacagca
tatgttagtgacatttaccattaaagaatacaattttcttgatatcccccaaactagacaattcaggct
attttggcatagctatcttaaaattcattatctcttgctgttttttttttttctttcagtaagttcattg
aggttcttacttcactttatttataattaagatttctatttggttcattttcaaatacacctgattctt
tttcttctatttttcagtaactctttttgaaaaatcagaagaattggagagtgatgttaaaaaccttaa
ttattaacttgcttaagtttagactctttaaaaattattgcataatttattgaactcccactagtttat
aagaaggatacattatttgctttggagctataagattaagcttgtttggtttaatttttttattgctgta
taattgacatacaccttattactttcaggagtacaacataatgatttgatatttctgtatcttgcaaaa
tgatcaccacaatgtctagttaacatccatcaccatacatagttacaaatttttttttctcatgatggga
acttttaaaatttactctcttagcaaccttcaaatgtgcaatacagtattattaactatagttgtcatg
ctgtacattacatccccgtgacttatttattttataactggaagtttgtacctggtttacttttaaagg
ttctgttctccctcataattttcataatgtaagtggagaggtggttttaatttcttctatgttaaaaaa
aactagacagacctaggagaaaagaaatgaattgttactttgtgcttttttaattacattcatttttaat
gggctttttcttgagcagttttaggtttacagagaaactgagcagagagtacagagtgttttcatatac
ccccttacccacctcaacccccagtttcccctattattaattacttgcattagtgtggtatatttgtgg
caattgatgaacccatattgacacgtcgttatcacccagagtccatagtttacattagagttcactcta
ggtgttgtacattctgtgggtttggacaaatgtataatgacatgtatccaccattatagtatcgtacag
agtagtttcactgctctaaaaatcctctgtgctttgtctgttcgacctctggcaaccactgatcttta
ctgtctctatagtttagtctcttccagaatatcatttagttggaatcatatagtatgtatgcttttcag
attggcttctttcacttactaatatgcatttaaatttcatccatgtttttttcatgacatgatacctaat
ttcttttagggctgaacaatattccattgtctggttctaccaccatttattgatccattcacctactga
aggacatcttggttgctcccaattttggaaattatgaataaagttggtataaacatctgtgtgcaggtt
tttgtgtagatgtaagttttcaattcattcattcattcatgtttgcaattgcaggattatataataaag
gtatgtttagctttgtaagaaaccttccaaaatggctgtatcattttgcattcccaccagcaatgaatg
agagtttctgttgccccacatcctcatcagcatttggtgttgtcagtgttttggattttagccattcta
ataggtgtgtagtggtatttcattgtttatttatttattggtgaggaagattggtcctgagctatcatc
tgttgccaatcttcctttctttgcttgaggaagattgtccctgaactaaaattcgtgctaatcttcctc
tatcttgtatgtgggattgccactagagcatggcttgatgagacgtgtaagctgtgcctggaatctgaa
ccatgaaccccaggctgccaaagcatagtacgcaaacttaaccactacgccaccggaccagcccctcat
tgttcttttaatgtgcaatttcctgatgacatatgatgttgagcatttttttataagtttatttgccatc
agtatatcttctttgatgaggtgtctgttcagttcttttgcccatttttaagttgagtctttgttttct
tattgttgagttgtaagagttctttgtatatcttggataccagtcctttattagatatattttgtaaag
attttctcccagtctctggcttatttttttcattctcttaacagtgtcttttgcagagcagaagtttta
attaattataaaatccaagttatcaatttttttctttcatagatcatgtttttttgtgttgcatctgagaa
gtcattgccaaactcacaatcccctagattttctcccattttgcttataggagtttatagttttgtatt
ttaaatttagatctatatccattttgagttaatttttatgaaaagtgcaaagtctgtgtctagcttcgt
tttctttgcatgtagatgtccagttcttctagcaccatttgttgaaaagactgtcctctccccactgaa
ttgcctttggtcctttgtcagagatcagttgactctctttgtgcggggttatttctgggccctctattc
tgttccactgatctatttgtgcattcttccactaatactacactgccttaatattaaaattacatccat
tttttgaataggaaatccactcagatggtacaaaatataagcagtacgaagagtatatagtgaagtata
gtgaagctgcttcctgccctgttcccccagctaccagattactttcccaaggctaccaccattatcaag
cgtaattttcattctttccgagttatttcactttttatcttatcctctctctcctctccccactcaccc
ctaacacaaatattaaaacactatgcacagtgttatgtgacttgcttttttttttcatttaagagtataa
ctaggaaaacatctcatatctgtacatctagccctgccttccttaaaaaagtctttattgtggcaaaaa
aacaccacataacgttacatttaccactttaaccattttaagtgtgcagttcagcagtgttaagtatat

ccacattgttgtgcagcagatctttcaaactataatctcgcaaaactgaaactctatacccattaaaca
ctaatgcccctctccctaacccttgtcaaccacttttctactttctgtttctatgattttgactatttt
agatatttcatatgagtggaatcataaagcatttgtccttttgtgactggtttatttccctgagcatga
tatcctcaaggttcattcatgttgtagcacatgaaaggattgccttctttttaaggctgcataatatt
ccattgtgtgtgtatgtatgtatgtatgcatgtatgtatgtatatatgtatgtatacatatcacatttt
ctttttatatatatgttctttattttattgaggtcataatagtttataacactgtgaaatttcagttg
tacattattatttgtcggttatcatatatatgtgcccctttacttcttttgtccacttcccaacccccct
cctgctctcgtaaccactaatctgttctctttgtccatatatttgtttatcttccacatatgagtgaaa
tcatttggtgttttctttctctgcctggtctatttagcttaacataatagcctcaaagtacatccatg
ttgttgcaaatgggacggttttgtctttttctatggctgagtagtatttcattgtgtatatatatatat
ataccacatcttctttatccattcgtcagtcgatgggcacttgggttgacttctgtatcttggctatag
tgaataatgctgcagtgaacataggagtgcataagtctctttgaattgttgatttcaaattatttggat
aaatacccaatagtgggatagccaggtcatatggtatttctttttttcttttttttcttttcttttaag
attggcacctgggctaacaactgttgccagtctttttttttttttttctgctttatctccccaaacccc
cctgtacacagttgtatatcttagttgcaggtccctctgtttgtgggatgtgggatgtggcctgacgag
tggtgccatgtccacgcccaggatctgaaccctgggccgccgcagcggagctcgcgagcttaaccactc
ggccatgggactggccccaaccaatcttcttcttttttttttttccttcttctctccaaagccccccagt
atattctagttgtgagtacctctggttgtgctatgtgggatgccatttcagcatggcctgatgagcagt
gccatgtccgcacccaggatccaaacctatgaaaccctgggccaccgaagcagagcacgtgaacccaac
cgctcagccacggggccagcccccatatggtatttctatttttactttttgataaatctccatgctgc
tttctatattagctgcaccagtttgcattcccaccagcagtgtaggagggttcccttgtctccacatcc
tctcccacatttactgtttttgtcttggtgattatagccattctaacaggtgtaaggtggtatctcat
tgtagttttgattttcatttccctaataattagttacgttgaacatcttttcacgtgcttattgtatat
cttctttgggaaaatgtctgtttgtatcctctgcccatttttgatcaggttgtttgtttttggttga
gttgtatgagttctttttatattttggagattaacctcttgtcagatatatgatttgcaaatattatct
cccagttggtgggttgtcttttcatttacttcatggctttctttgccttgcagaagcttttagtctga
tgtaggcccatttgtttattttttcttttgtttctcttgcctgaagagacatagtatttgaaaaggtcc
ttctaagactgatgtcaaagagtgcactacctatgttttcttctaggagttttatggtttcacatctta
cattcaagacttcactccagtttgggtgattttgtgtatgatgaaagataatggtctactttcattctt
ttgcatgtggctgtccagttttcccaacaccatttattgcagagactttcctttctccattgtatattc
ttggcacctttgtcaaagattagctgtccatagatgtgtggtttctttctgggctttcaattctgttg
cattgatctgtgtgcctgtttttgtactagtaccatgctgttttgatcactgtagctttgtagtatatt
ttgaagtcagggattgtgatgcctccagcttcattcatctttctcagcattgctttagaaatttggggt
cttttgttgccccatatgaattttaggattgtttgttttatttctgtgaagaatgtcattaggattctg
actgggattgcgttgaatgtgtagattgctttaggtattattggcattttaactatgtttactcttcag
atccatgagcatggaatatctttccctttctttatgtcatcatctatttctttcaatgacgtcttgtag
ttttcagtgtatagatctttcacctccttggttaaatttattcctagatatttattctttttgttgcg
attgtaaatgggattgtattcttgagttctctttctgttagctcgttgttagagtatagaaatgcaact
gattttgtaagttgattttgtaccctgcagctttgctgtagttcttgattagttctaatagttttcca
atggattctttagagttttctacatataaaatcatgtcatctgcaaacagggagagtttctcttctttc
tttctaatttggacaacttttatttttcttgcctaattgctctggccaaaacctccactactatgttga
ataagagtggtgagagtgggcacccttgtcttgttgctgttctcagagggatggctcttagtttttcct
tgttgagtatgatgttggctgtgggtttgtcatatatggtctttattatgttgaggtactttccttcta
tacccatttcattgagagtttttattgtaaaagggtgttggatcttatcaaatgttttctctgcatcta
ttgagatgatcatatgattttattcctcatttgttgatgtggtgtatcacattgattgattggtgga
tgttgaaccgtctgtgcatccctggtgtaaatctcacttgatcatggtgtataatctttctgatgtatt
gctgtattcgggttgccaatattttgttgaggtatatcacatttctttattcactcatctgtaaaagg
acacttggattgtttctccctttggctattgtgaataatgctaccataaacatcagtatcaaatatctc
tctgagaccctgctttcttttgttttaggtatgcatccagaagtggaaatgttagaccatatggtaatc
ctatttttaactttttgaggaacctccatactgttttccacagcagccacaccatttacatccccacc
aacagtgcataagtgttccctttcaccacatcctcaccagcatttatctcctgtctttttatcatagcc
attccaacaggcatgaggtaatatctcattgttgttttgatttgaatttctcttatgatttgtgatgtt
gagcatcttttcacatgtttgttggccatctgtatatcttctttggggaattgtctattcaagtccttt
accaatttttttaattgagttatttgtttttttggtgttaaattgtagaagttccttatatattctggatg
ttaaccccttatcaatatatgatttgcaagtatttctcctattccataggttgtctttttttttttta
aagattggcaactgagctaacaactgttgccaatcttctttctttttctttccaaagacccccactaca
tagttgcatattctagttttgagtgcctctcgttgtgctgtgtgggatgctgcctcagcatggcctgat
gagcggtgccatgtctgtgcccaggatccaaactggcggaaccctgggccatggaagtagagcacacaa
acttaaccactcagccagggggccggccccaataggttgtcttttcattctgttgatagtttcctttga
tgtgcagaaatttttaagcttgatatacagtcatgcattgcttaaagatggggaagtctgagaactgca
tcatgtggtggtttcctagttgtgcaaagatcatagagtgtacttacacaaacctagatggtatagcct
actacacacctaggttatatggtattaatctcatgggaccactgttgtatatgtggtccgttgttaact
gaaacgttgttatgtggcacacgactgtagtctcatttgtctgttttacttttgttgcctgtgctttttg

gtgtcatatccaagaaaccattgtcaaatctaatgtcctgaatctttccccctatgttttcttctagga
attttataggtttaggtctttaatccattttgaggtaatttttgtatatggtgtaagttgagggtccaa
cttcactatttgcaagtggatatccaattttcctcacaccatttgttgaagagactgtcttttccccat
tgtgtagtcttggcatccttgttgaaaattatttggccatatatgcaaaggtttatttctgggctttct
tttctgttccattggtttatatatctgtctttatgccaatgccacatcatcttgattactgttgctttg
tagtatttttttgaaatcaagaaatgtgtagtcttcaactttgctcttcctttgtaagattgtattggct
attcagggtcccttgagcttccatgtgaactttaggatttttttttttcctatttctgcaaataatgata
ttgggattttgatagggatttcattgaatttgtaggtccctttggctagtatggccattttaacaacat
taagtcttccaatccatgagcatggcacgtctttccattcatttgtactttcttttaggaatagtttct
agatttcagtgtacaaatcttttgcctccttggttcagtttattccttagtattttatcctttctgttg
ctatagtaaatgagatttgtttaaaaatttttttggattgatcattgttagtgcatagaaatgcaaat
tatttttgaaaaattttttatagtaaggtgctatatggtattctgttgtatgcatagcacaattcactta
accagtctcctattgaggaatatctatgttatttacagagctttgatgttacaaacaatgctgcagtgc
acagtcttgtgaacatctcattttcacatatatctgttggataaattcctagaagttaaatttctgga
ttaaaggatatatgcattgaaaaatttcatagatattgccaaagcattatgattttgacataaaacagt
cagaatttgtgatggtatttattgcaaattatgagagcatctattttctcctccaatttgcttttttt
ttttttttttaaagatttatttttttcctttttctccccaaagcccccggtacatagttgtatattct
tagttgtgggtccttctagttgtggtatgtgggatgctgcctcagcgtggctcaatgaatggtgccatg
tccgcgcccaggattcgaactgacgaaacactgggccgcctgcagcggagcgcgcgaacttaaccactc
tgccacggggccggcccctccaatttgctcttttaagggtaatttactttgtctttcttccaaagcaga
ttcacaaattacacatttatggtcaagagtccaagaaagggttttatgttgatttctgtggcaatttga
tgaaaaatatgaaagagatatgggccagtgaactttttctaaaatatagctgaaggagactcattgaga
atcatctactaaaagtgatatatgtacaataatatatcagtaaaactttgttagtcatcctttattgga
gttgtctttcttatgattcacaaattttaaaaattctgcaattatcatttttgttattaagatgtttctc
ttttgtggcatttgtcatttaaggctgtttagtgttaagaagtcttgtcagcaagaactagtttaaaag
tcatgtgctgtcatgtaaaggagtggctctcctgtcaaatttattgcatttcccaaggaggaaaatata
acttctgtaaattgtaggtttaagtgacattcaatgtatggaagacaagaataaaatcagtataccaga
tacagaaggctagtttgtgtatttggcaatggtacttgaatacttgacagaggacttgaaatgtgtaaa
agcttctcttgattaaaaagttgccggagtctatcagattatcaaagtgttccattcaagaaaggctca
tcacttgctattccagtagaaagcagcaaccctgtcacctaaacacaacaggaggtcttgcaaatttca
agaaaagtaaatggaatggactaaaacatgtatggcggaataaaattggaaataaaatttgaagccatt
ccattttagagaactgatttgtgacttgcagagattaccaatcgacaattaaattatagaacatgaaag
ttggggaggcttttagagctcatccagccaaacctctgtaatgaggagaaaatagatgtagattggttg
gtctgtgagctaaatatcacatcagactggggaaaaggtcgatgtgtatgtgtattccaaatggcagtt
ttggatttattcaatattgaaagaattgtatgcagatctcaagttggtcttatgttatacacacacaca
cacacacacacacacacacacacacagaggcatacattggggatattgtgggtttggttccagacca
ccacaataaagcaaatatcacaataaagcaagtcacaggaacttttttggtttcccagcacatataaaag
ttatgttgacactatgctatagtctattaagtctattaatattaagtggcattatgtctaaaaatatat
acatgccttaattaaaaatactttatcactaaaaaatgctaaccatcatctgagccttctgtgagtcat
aatctttttgctggtggagggtcttgcctcaatgctgatggctgctgactgatcagggtagttactgct
gaaggttggggtggctgtggcaacttcttaaaataagacaacagtgaagtttgccacattgattgattc
tttctttcatgaatgatttctctgtagcatgtgatgctgtttgatagcattctacccacagaacttctt
tcaaaattggaatcaatcctctcaaaccctgctactgctttatcgacaaagtttatgtaatatcctaaa
ttctttgttgtcatttcaataatcttcacagcatcttctccaggagtagattccatctcaaaaaaccat
cttttttgctcatccgtaagaagcagctcctcatctgtgaaagttttatcatgcgattggagcaatata
gtcacatcttcaggttccacttccaattctagttctcttgctatttctaccacatctgccgctacttcc
tccactgaagtcttgaacccctcaaagtcatcctgaggtttggaatcaacttcttctaaactcctgtta
atgttgctattttgacctgttcccatgaatcacaaatattcttagtggcatctggaatggtgaatcctt
tctagaaggtttgcaatttactttgcccagggccatcagtggagtcactaactatggcagctatagcat
tacaaaatgtatttcttcaataataagagttgaaagttgaagttactccttggtccatgggctgcagaa
tggatgttgtgtgaggaggcatgaacacaatattaatctcgttgcaatctccatcagagctcttgggtc
accaggtgcattgtcaataagcagtaatattttgaaagaaatcttttttttctgagcagtaggtctcaat
ggtgggcttaaaatattgactcaactatgttgtaaatagatatgctgtcatccaggctttgttgtttga
tttatagagcttagacagagtacctttagcataattcttaagggtcctaggattttcagaatggtaaat
gagcattggctttaacttgaagttaccaggtacattagcccctaacaagagagtcagcctgtcctttga
agctttgaagcaaggcattgacttttcctcgccagttatgaaagtcctagatggcatcttcttctaata
taaggctgttttgtctacattgaatatctgttgttttatcctggttatatcttctggagaacttgttgc
agcttctacgtcagcacttgctgcttcaccttgcactttcatgttatggagatggtttctttccttgaa
actcatggaccaacctctgctagcttcagattttcttctgcagcttgctcacttctctcagccttcag
aggattgaagagctaggaccttgctccgcattaggctttgggttaaggggatgttgtggctggtttgat
cttctatccagactactaaaactttctccatatcagcaataaggctgctttttttatttgcgtgttcact
gaagtagcacttttaatttccttcaagaacttttcatttgcattcacaacttggctaattgtttggcat
aagaggcctagctttcagcctatcttggcttttgatatgtcttcctcactaagcttaatcatttctagg

ttttgatttaaagtgagagacatgcgactcttcctttcacttggaacacctggaggccattgtagggtt
attaattggcctaactttaatattgtgtctcagggaatagggaggcctgaggagcgggagagagaaggg
aacttgctgtctgaacacacatctattaagtttgctgtcttatgtaggtgaggttcgtggtgccctaaa
acaattataatagtaacctcaaagaccactgatcacagatcacgataacaaatatagtaataatgaaaa
catttgaaattttgcgagaatttccaaaatgtggtgcacaggcatgaagtaagcaaatgctgttggaaa
aatggcaccaacagacttgctcaaggccacaaaccttcaatttgtaaaaagtgcaatgtctgagaagag
caataaagtgaagtgcaataacacgaggtatgtctgtgtctatgtttatgattttttttttaaaagattt
tctttttcctttttctccccaaagaccctggtacatatttgtattatattcttcgttgtgggtccttcc
agttgtggcatatgggacaccgcctcagcgtggtttgatgagcagtgccatgtctgcgcccaggactcc
aactaacgaaacactgggccacctgcagcggagcgcaggaacgtaaccactcggccgcgggtttatgtt
tttaagcattgtggcatgctgactattatatataaaacatctaggaaagcaaagaacttgatgataacc
tggaatataagaaaaaatatacaatatggtcatatagagtagtgattaagagtgcagtgtcaggtctga
atttcagttccatgactgtgggactttgtgtcttggtttcctcatctggaaaatgtagctaacaaacgt
ttatctgcctcaaagggcataaggattacatgagataacccagataaaggcttagtatagtaatatact
aatattatagtattcatatatagtatgattatatagtaaaatattattgttttgtcaataagtccaaga
aaagaaggcgggtcaaaaagcactttccgaataaagtcaaaatacgtcactagcactttcaggtcaatg
ggactgaactggacccccacgacaaggtagacgggatcaaatgacgtagaccgccttagcaacaggcc
cccaaagaaggtgaatctccgccgccaataagcgcctgcgcctcggcggagggagccaatggacgacgg
gaagcggaagagggaggccgcgccaattctcctgcctgagcctcggcccaagaaaatggcggcggcggc
ggcgtcgcttttgtttccgcggctc

>elephant
caccagtaattatcaatgaaccttttcatattgaactaaggtttaggttgtcatgattgctacttgaat
tcatatctactgacagaaaatagggttgttattgcttacaacaggtaaagaactattcatttttttccat
catcatgttatattctcattgtaataaatttggaaaaaaattgaaaggtagaaagtagaaaaaaacaac
caggcatgttacctatagatagctaatcttttcattttgggatgtatcctccaagtctttcttataata
acacttttttcttttaaatggaattttaaaaatgaatactttataaacatatttctgtaagtcgaggtga
tgctttaatgccttgtgacgttccacattatgaggcacgtgttgactttttttataacaaaatatgtgat
agccttgattctcgtaatttgaaaaatacagcattactagaataagttggtatatgttaatcacattta
catttttttatgtacagtatttttgatgttctccaaaagtagacaacctggctgattttgtcagagttat
tttcaaaattcactgtctcttgtttttttcactagtaagtatattgaggtttttacttctagcactctat
ttatataacacgtttttgttccttttcaaatccacctggtatttttttcttattttccctttaataatac
tttttgaaagatcagaaaattaggatagtcgtgttaaacaccttaattataaaattgccttaattttag
gatttttaaaaatcattgcatcattaattgaattccaatggtttataagaaggaaacatcttgtttgga
gcatgatattaagcctgtttgatttactgttgaagttgttttctcccccataattttcttaaggagtga
agaggtggtttttggttgctttatgttaaaaaaaaaatagaaaacaagacaaacctaaaagaaaaattga
attgttactttatgatttgttcattacattcattttgtcaataggaaatacactcacatggtacaaaat
gtcaacattataaaagggtgtacggtgaaaagtttcactcttgcgctgtcccacatctatcaagttacc
ttctcagggcaaccactattatcaagtttttttttttctttctctcccctaacagaaatattaaaatac
tgtacacaactgtctacaacttgcatttatcatttaacaatatacctaggaattcattccatatctgta
cgtcttgactttcctatttacggagctatataacattctgttgtagggtgtaacataattatttatcca
gtctcatattgagaaacatctatgttctttccaaagttttgactttacaaacaatgcagtagtgaacgg
tcttgtagacatctcattttgcacatgtagaatgtataaactagtgccatacgtaggataaattactag
aagttacattgctgggtgaaaggatgtacgcatttgaaatttttcacagacgtgggtaaatcattttga
ttttgacataaaatagtcaatggttgtgaagtgtttattaaaaatgatgaaagttcttatcttctcatg
gggtttgctctcttaaagttaatttactttttctttcttccaaagcaaataatcaaattcacaaatcac
acattttatggtcagcagtgctagaaagggttctatgttgatttctgtggcaatctgaaggaaaatgtg
aatgatatgtgtacactagtgatttttttctaaaatacaattgaaggaaacccattgagaatgatgcat
gaaaagtgatacacccacattttatatcactaacattttattaaaacaaaccacaaaccaagcccagtg
ccgtcgagtcgtttccgactcatagcgaccctataggatggagtagaactgtcccatagagtttccaat
gagcgcctggcagatacgaactgccgaccttttggttagcagccgtagctcttaacctctacgccacca
ggaacattttattaaccattatttaatagcaaaggaggtctctctcttatgattcacaaaaatagttat
tgtgtatttcaaaaattctgtgattataattttattaagatgtttttctacttgtgaaatttgtcattt
aaagctgtttagtgttaagaaatcttgtcaccatgaactagtttataaataatggaaatatataaatta
gtaaccattagtgctttagaaagaagttaatgccaactatttctacaataaaatttccaatcgatgtta
agattttggaccgttcactatttttacccatagtgaatctgtttgatagctagtatatttgtgtgctgc
cttgtaatggaatggatctcctttcaaagtaactgtatttaccaagtactactgtaaattgcatgttta
aatgatatttaatgtatggaataagaagaataccagtatattagatacagaagattgctacgtcaaaga
agccagcaacggcacttcaacatttgaaaatgacttcaaatctgtacaggcttcagttgcttaaaaagc
tccctagtccatcagattatcaaggtgactcattcaaaaaaggctaatttcttgctattccagtagaaa
gcagcaacctaaccacaaccggggttctcgcaaatttcaagaaaaacaaataaaatgaactaaagcacg
catggtagaataaatttcgaaataaaaattgaagccattttattttagagagctgatttgtgagttgca

gatgtagttaccaacgtagaaacaaattatagaatatgaaagctggaaagactttcagatttcatccag
ccaaacctcctccgtaatgaggtggaaacagatctagaatggttggtttgtgagctacacttcaggatg
gggaaacggctgggctgcggtgtgtgtgtgtgtgtgtgtgtgtgtgtgcgcgcgcgcgcgcgcgc
gcgtgcgttctaagggacagtctagattttactcaatattgaaagaattgtacgtagatctcaagttag
tcttatgtcatatatgtatatatatatttaagcatgctggggtgataagtagtagatatgaagcatctt
aggaaagaaaagaacttgataacttggaatataggcatacaggaaagaaaacatttatacaggattgtc
atactgattagcggttaagacgactcctctgaatctcggctccactaccgtgggacttcgtgccttggt
ttcctcatctggaaaatgtaggtaataagagtatttgcctcaaagggtataaagattgcacgaggtaat
ctgggtaaggattagtatagtgctggatatacagtaaactctcaataaatgtaaattgttttgtcagta
tgtcagtgaaaagacggaccaaaagagctttttcataataaagtcaaaatacgccactaacactttcac
gtcaatttggactgaactggacccccagcgaaaaggtggaggggatcaaatgacgtcgaccgccttagc
aacaggcccccaaaggaggtgaatttccgtagccaataagactctgcgctgtggaagaggtagccaatg
gatgacggaaagaggaagaggtaggacgcgccaattctcttgcctgagcctcggcccaacaaaatggcg
gcggcggcggcgtcgctttgtttccgcggctc

Figure 3a

>elephant
catactgattagcggttaagacgactcctctgaatctcggctccactacc
gtgggacttcgtgccttggtttcctcatctggaaaatgtaggtaataaga
gtatttgcctcaaagggtataaagattgcacgaggtaatctgggtaagga
ttagtatagtgctggatatacagtaaactctcaataaatgtaaattgttt
tgtcagtatgtcagtgaaaagacggaccaaaagagctttttcataataaa
gtcaaaatacgccactaacactttcacgtcaatttggactgaactggacc
cccagcgaaaaggtggaggggatcaaatgacgtcgaccgccttagcaaca
ggcccccaaaggaggtgaatttccgtagccaataagactctgcgctgtgg
aagaggtagccaatggatgacggaaagaggaagaggtaggacgcgccaat
tctcttgcctgagcctcggcccaacaaaatggcggcggcggcggcgtcgc
tttgtttccgcggctcctgcggcggtggcagtggtagtggcctttaagct
gtggggaggctccatcacaagctacagggacgaataaggctccagtgcat
tgtatcgtccccaggcgacgggtagcgcagagtggcaccctacgggtcgc
agaagccgacaaggcgttgaagcgaaaacatgaccgctgagcccatgagg
taaaacgcaaccccgtctcaatagccccagcaacctgggcctaggcac

>human
gtgcctgggcccagaccgctggggcccatgagacggggttgcgttttacc
tcatgggctcagcggtcatgttttcgcttgaacgccttgtcggcttctgt
gattgctgggcgccgatctgcgctcccccgcgcccggttacgatagaaat
gcactggagtcttagtcgtcactgtagctgctgctggaacctccccacag
ctcaaaggccgctaccactgccaccgccgcaggagccgcggaaacaaagc
gacaccgctgccgccgccattttgttgggccgaggctcaggcaggagaat
tggcgcgtcctccctctttctctttccgtcagccattggctacctcctcc
aaagcacagagtcttattggctacggaaattcaccttctttgggggcctg
ttgctaaggcggtcgacgtcatttgatcccctccaccttttcgcaggggg
tccaatttagtcccaattgacctgaaagtgctagtgacatagtttgactt
tattttgaaaatgctcttttggtccaacctctttttttctttgacatatta
acgaagtaataacagcaacctacatttattgagagtttactatatgctcg
gcactacactaatcctttatccgggttatctcatttaatctttacactct
ctgaggtagacaaacctacattttccagatgaggaaaccaagacacaaag
tcccacagtagtggagccgagattcaccagacactcttaaccactaatct
gtatggccatcctatgtaagtattttctttcctatatgctaggctatcat
c

>dog
gcgcataggcccggaccgctaggacccgtgagacggggttgtattttacc
tcatgggctcagcagtcatgttttcacttcaacgccttgtagcttcaacg
accgtaggagcgccgctttgcgctctccggccctgggttacaacagaaat
gcaatggactcttagtcgtcactatagctggtgctggatcctccccacag
ctcaaaggccgctaccactgcccccgccgcaggagccgcggaaacaaagc

```
gacgctgccgccgccgccattttgttgggccgaggctcaggcagaagaat
tggcgcgtcctccctcttcttctttccgttacccattggctacctcttcc
acagctcagactcttattggctactgaaattcaccttctttgggggcctg
ttgctaaggcggtctacgtcatttgatcccctccaccttttcataggggc
tccgattcaatcccaatgacctgaaagtgctagtgacgtattttgacttt
attctgaaaatgctttttgatccattcgcttttctttgacataatgacga
aacaataacagcaatttacatttactgagagtgtagtatatacccaacac
tatgctaatcctttatccggattatctcatgtaatccctattctctttga
ggcacacagacccttgttaactacattttccagatgaggaaaccaaaaca
caaagtctcacagtagtagagccaagattcagaccagaaaccacattctt
aaacactaatctgtatgaccatcctatatattttcttttctatgttctag
a
```

>mouse

```
cgccggggcccgtgacaagggcttgcgttttacctcatgggctcagcggt
catgtttttggctcggacggccgcttggcttggcaaccccttgggtaccg
ttctgcgctcctcggcgccagtggctgaacgaaaagtactgggatcttag
tagtcaccgcagatagtgctggatcgtcctcccagctcagagtccgctac
cgctgctgctgctgctgctgctgctgctgcaagagccacggaaacaaagc
aaggcctatgccgacgccattttgttgggccgaggcttgggcaggagaat
tggcgcgtcctccctcttcctctttccgtcatctattggctacctctgcc
agagctcagtgtcttattggctacggaaattcaccttctttgggagcctg
ttgctaaggcggtcgacgtcatttgatcccctccacctcttcttagtggg
gtccagttcagttactgctgacctcaaagtgtcagggatgtatcttgaca
ttatcctgaaaatgcgctcttggtccaacctctctacttccacataattg
gtgaaacaatgacaccccatttacgtttattgagaatttactatatgtcg
accactacacagatcctttatcttaatctcatgcttgaaccatccaaagt
aggcagacctaacattttaatgggtgaggataccaatgcacaagttccac
attcagccaaacatccatccttgcggggagggtggacatacattcttatg
ctgttttaaacccaaggctttgtgcatgttaggcaaatactctatcattg
a
```

>horse

```
gggcgcggaggcccggcccgccgggacccgggagacgggcttgttttacc
tcatgggttcagcagtcatggtttcgcttcacggcttgtcggcttcagcg
agcggaggggcgccgctctgcgctcccggcgcccggttacgacagaaatg
cactggcctcgtcgtcgtcaccgtagctgctgctggatcctccccacagc
tcaaaggccgctgccgctgccaccgccgcaggagccgcggaaacaaaagc
gacgccgccgccgccgccatttcttgggccgaggctcaggcaggagaat
tggcgcggcctccctcttccgcttcccgtcgtccattggctccctccgcc
gaggcgcaggcgcttattggcggcggagattcaccttctttgggggcctg
ttgctaaggcggtctacgtcatttgatcccgtctaccttgtcgtgggggg
tccagttcagtcccattgacctgaaagtgctagtgacgtattttgacttt
attcggaaagtgcttttttgacccgccttcttttcttggacttattgacaa
aacaataatattttactatataatcatactatatatgaatactataatat
tagtatattactatactaagcctttatctgggttatctcatgtaatcctt
atgccctttgaggcagataaacgtttgttagctacattttccagatgagg
aaaccaagacacaaagtcccacagtcatggaactgaaattcagacctgac
actgcactcttaatcactactctatatgaccatattgtatattttttctt
a
```

>wallaby

```
GTTGTTGGCCTTAACACATCACACTGTTAGGGAAGGGGACGGCAATGAACCGTCAGGATG
AAGGGAAGACACGTCATTACTACTTTCAGGCCACTGTAGACTGCTTTGGATCCTTTTGAA
AATGGGTGGGGCAACCAAGTGACAAGGCCAGCCCTAGCAACAGGCCCCAAAAGATGGTAA
ATTGTGGGAGCCAATACGAATCTATCCCGGCCAAGCGTTAGCCAATGGGCGGTGTCGCAC
GGCGCGGAGGGAGGACGCGCCAATTCTTCGGCTCGAGCCTCGGCCCAACAAATGGCGGCA
GCGGCGGCGGCGGCGGCGCTTTGTTTCCGCGGCTC
```

<u>Figure 3b</u>

CR3

>armadillo
CTTCAAAAAAATAATAAAATAAAATGAAACAATATTGATCTATGGTTTAT
GTTGCCATGATTGCTATTAATGTTTGGATTCATATGTGCTGGCAAAATGGA
TAGCCTTATGTTTCTTACAACAGTTAAAAAATTATTTAATTTCCCCATCATC
ATAA

>elephant
CACCAGTAATTATCAATGAACCTTTTCATATTGAACTAAGGTTTAGGTTGT
CATGATTGCTACTTGAATTCATATCTACTGACAGAAAATAGGGTTGTTATT
GCTTACAACAGGTAAAGAACTATTCATTTTTTCCATCATCATGA

>mouse
TATTAGAATTTAATGAGAACATCTGTTAAAATAATGAGCTGAGGTTTATGT
CAGCATAATCCCTACTTGCTTCATATTTGTTGCTAGAAAATGGATAATCCT
TTTGTTACTTGCAACAATTAAAATTAATTTCCCCCATCGCCTTTA

>human
TATTGAAATCTAGGAAAGTACCTTTTAAAATAATATTGAGCTAAGATTTGT
GTGGAGATAATTGCTCTCTGAATTCATATATGCTGGCAGAAAATAGGTAG
CCCTGTTACTGCTTACAACGATTAAAATTATTTCATTTCTCCCATCATCTTT
A

>horse
TATTTGAATTTTGGTAGAGTACTTTTTAAAATAATATTGAGTTAGGGTTTA
TGTTGGCATAATTGATGCTTGGATTCATACTTGCTAGCAGAAAATGGATGG
TCCTATTGTTGCTTACCACAATTAAAATTATTTAACTTTCCCTACCAGCTTT
A

CR2

>armadillo
CAGTGATGTCTGTTTGGTGGCAAATGTGTTGTCTCCTGAGTGGGTCTTCTT
TCAAATTAACTGCTTTGACCCTGTACATTTTGTGTTTAGGTGATGTTTAATG
TATGAAAGACAAGAAGAAAATTTATAGAGCAGATGTAGAAAGCTAATTTC
TACTGTCTGCAAGGGAATCCAACATTTAAAAGATGAGTTGAAATGTGTAC
AGGGTTCAGCTGATTAAAAA

>elephant
TAGCTAGTATATTTGTGTGCTGCCTTGTAATGGAATGGATCTCCTTTCAAA
GTAACTGTATTTACCAAGTACTACTGTAAATTGCATGTTTAAATGATATTT
AATGTATGGAATAAGAAGAATACCAGTATATTAGATACAGAAGATTGCTA
CGTCAAAGAAGCCAGCAACGGCACTTCAACATTTGAAAATGACTTCAAAT
CTGTACAGGCTTCAGTTGCTTAAAAA

>mouse

TAGCAAGGATATTTGTTGTGATGAAATAGATCTTTCAGGTTAACTTCATTT
CTCAAGGAGGAAGAACATGGCTTCTGTAAATTGTGTTTAAGTAATATTTA
ATATATTAAACACAAAAGAAGAACATTATTGAACAACAGAAGATTGATTT
CCACAGTTGGTAGTGGATCTTGAACATTTAAAAATTATTTGAAATGTTGCA
AGTTTCTGTTCATCAAAAA

>human
CAGCAAGCACATTTCTGTGCTGCCTTGTGATGGAGTACATCATTCAAATTC
ACTGCATTTACCAAGGAGAAAAAAATGACTCCTGTAAATTGTATGTTGAA
ATGATATTTAATGTGTACAAGACAAAATCAGTATAGCAGGTACAGAGGTC
TGGTTTCTATAATTGCCAACTGAACTCAAACATTTGAAAGATATAATTGCA
ATGTGTACAGGCGTCTGTTGATTAAAAA

>horse
CAGCAAGAACTAGTTTAAAAGTCATGTGCTGTCATGTAAAGGAGTGGCTC
TCCTGTCAAATTTATTGCATTTCCCAAGGAGGAAAATATAACTTCTGTAAA
TTGTAGGTTTAAGTGACATTCAATGTATGGAAGACAAGAATAAAATCAGT
ATACCAGATACAGAAGGCTAGTTTGTGTATTTGGCAATGGTACTTGAATA
CTTGACAGAGGACTTGAAATGTGTAAAAGCTTCTCTTGATTAAAAA

CR1

armadillo
TTTCTATCAGATTATCAAAGTGATCCATTTTAAAAAGGCTCATCTCCTGCT
TTTGCAGTAGATAGCAGTAACCCTATCACCTAACCACAATAGGAGCTCTT
GAAAATTTCAAGAAAAACAAATGTATTGAATAGGATTGAAGTAAAGTATA
CATGGAAGAATAAATTTGGAAATAAAATTAGATGCTATAGATCTTAGAGG
ACTGGTTTTCACTTGCAAAACTTGCTACTAATGTAGAATATAATTTTAGAA
CATGAAAGCTGTAAATACTTTGAGAGATCATACAGCCAAAACTCCTCTGT
ATTGAGGAAGAAACAGACCTAGAATGCTTAGTTTATGAGCTAGATAACAC
ATCAGAAAGGAGAAATGGCTTGCGAGCCTGTGCGTTCCAAGAGGCAGTTT
ACAGTTTATTTTGTATTAAAAGAATTGTATGAAGATCCCAAGTTTTATGTC
ATATAT

elephant
AGTCCATCAGATTATCAAGGTGACTCATTCAAAAAAGGCTAATTTCTTGCT
ATTCCAGTAGAAAGCAGCAACCTAACCACAACCGGGGTTCTCGCAAATTT
CAAGAAAAACAAATAAAATGAACTAAAGCACGCATGGTAGAATAAATTT
CGAAATAAAAATTGAAGCCATTTTATTTTAGAGAGCTGATTTGTGAGTTGC
AGATGTAGTTACCAACGTAGAAACAAATTATAGAATATGAAAGCTGGAAA
GACTTTCAGATTTCATCCAGCCAAACCTCCTCCGTAATGAGGTGGAAACA
GATCTAGAATGGTTGGTTTGTGAGCTACACTTCAGGATGGGGAAACGGCT
GGGCTGCGGTGTGTGTGTGTGTGTGTGTGTGTGTGTGTGCGCGCGCGCGCG
CGCGCGCGCGTGCGTTCTAAGGGACAGTCTAGATTTTACTCAATATTGAA
AGAATTGTACGTAGATCTCAAGTTAGTCTTATGT

human
AGCCTATCAGATTTTCAAAGTGTTTCATTCAAAAAAGGCTCCCCTCTTGCT
ATCCCAGTAGAAAGCAGCAACCCTGTCACCTAACCCCGACAGGAGCTCTT
GCATATTTCAAGAAAAATAAATAGAATGAACTAAAGCATGCATGGCAGA

ATAAATTTGGAAATAAATTTCGAAGCGATTAGATTTTAGAGGACTTGTTTG
TGATTAGCAGAGATTACCAATGTAAAATCAAATTATAGATCATAAAAGCG
GGAAAGGCATTTACAGATCATCCAGCCAAGCTTCCTCTGTAATGAGAAGG
AAGCAGATCTAAATTGATTGGTTTGTGAGCTAAACATCACATCAAATTGG
GGAAATGGCCCGTGTGTGTGTGTGTGTGTGTGTGTGTGTGTGTGTGTATTC
TAAGAGACACTTTATTCAATATGGAAAGAATTTTATGC

horse
AGTCTATCAGATTATCAAAGTGTTCCATTCAAGAAAGGCTCATCACTTGCT
ATTCCAGTAGAAAGCAGCAACCCTGTCACCTAAACACAACAGGAGGTCTT
GCAAATTTCAAGAAAAGTAAATGGAATGGACTAAAACATGTATGGCGGA
ATAAAATTGGAAATAAAATTTGAAGCCATTCCATTTTAGAGAACTGATTT
GTGACTTGCAGAGATTACCAATCGACAATTAAATTATAGAACATGAAAGT
TGGGGAGGCTTTTAGAGCTCATCCAGCCAAACCTCTGTAATGAGGAGAAA
ATAGATGTAGATTGGTTGGTCTGTGAGCTAAATATCACATCAGACTGGGG
AAAAGGTCGATGTGTATGTGTATTCCAAATGGCAGTTTTGGATTTATTCAA
TATTGAAAGAATTGTATGCAGATCTCAAGTTGGTCTTATGT

dog
TGTCTATCAGATTGTCAAAATTTTCCATTGAAAAAAGGCTCACTATTTGCG
ATTTCAGTGGAAAGCAGTAACTCTTTCACCTAAATACAATGGGAGCATTTT
CAAATTTCAAGAAAATTAAATGAACTAAACTATATATGTCAGAATAAATT
TGGAAATAAAATTAGAAACTATTCAATTTTGAAAACTAATTTGTGACTTGC
AGACATTAGTAGTGGACAATTAAATTAGAGAACATGAAAACTGGAAAGG
CTTTTTAGAGATCATTCAGCCAAACTTCTGTAATGAGGAGAAAATAGACC
TTGATTGGTTGGTTTGTAAGCTAAACTTTACATCAGATTCGGAAAATGATC
GTGTGTGTTTCAGTTGGCAGTTTCGATTTTATTCAGTATTGGAAGAACTGC
ATGAAGATTTCAAGTTGGTTTTATAT

mouse
AACCTAGTCTGATTGTTGGAATGCATTGTTTGAAAAGAGACTCTTCTCTTG
TCATTCTAGTCCAAAACTGCAACCCTGACATCTAAGCATGACCAAAACTG
TTGCATATTTCAGGAAAAATAAACAGAATGAACAAAATAAAGGGAATGA
ACATGTGTTCCAGAATAAATGTGGGCACAAAGTTGGAAACTTCAAATTTA
GAAAACTTATTTGTGATTTGCAGAGATTATGAATGAAAAATGAAATGAGG
GGGAAACGATTTGGATCAGTTGGTAAGTGAGATATACCCCCAATCATATC
AGATTGAACTAATGGCTGACCTGTGTGTGACAATTTCAAGATTGTTCAATA
TCAAAAGAATTAGATGTCAATGTCCAGTTGATCATATAT

Promoter

elephant
GTTTTGTCAGTATGTCAGTGAAAAGACGGACCAAAAGAGCTTTTTCATAA
TAAAGTCAAAATACGCCACTAACACTTTCACGTCAATTTGGACTGAACTG
GACCCCCAGCGAAAAGGTGGAGGGGATCAAATGACGTCGACCGCCTTAG
CAACAGGCCCCCAAAGGAGGTGAATTTCCGTAGCCAATAAGACTCTGCGC
TGTGGAAGAGGTAGCCAATGGATGACGGAAAGAGGAAGAGGTAGGACGC
GCCAATTCTCTTGCCTGAGCCTCGGCCCAACAAAATGGCGGCGGCGGCGG
CGTCGCTTTGTTTCCGCGGCTC

mouse

GTTTCACCAATTATGTGGAAGTAGAGAGGTTGGACCAAGAGCGCATTTTC
AGGATAATGTCAAGATACATCCCTGACACTTTGAGGTCAGCAGTAACTGA
ACTGGACCCCACTAAGAAGAGGTGGAGGGGATCAAATGACGTCGACCGC
CTTAGCAACAGGCTCCCAAAGAAGGTGAATTTCCGTAGCCAATAAGACAC
TGAGCTCTGGCAGAGGTAGCCAATAGATGACGGAAAGAGGAAGAGGGAG
GACGCGCCAATTCTCCTGCCCAAGCCTCGGCCCAACAAAATGGCGTCGGC
ATAGGCCTTGCTTTGTTTCCGTGGCTC

human
ACTTCGTTAATATGTCAAAGAAAAAAGAGGTTGGACCAAAAGAGCATTTT
CAAAATAAAGTCAAACTATGTCACTAGCACTTTCAGGTCAATTGGGACTA
AATTGGACCCCCTGCGAAAAGGTGGAGGGGATCAAATGACGTCGACCGC
CTTAGCAACAGGCCCCCAAAGAAGGTGAATTTCCGTAGCCAATAAGACTC
TGTGCTTTGGAGGAGGTAGCCAATGGCTGACGGAAAGAGAAAGAGGGAG
GACGCGCCAATTCTCCTGCCTGAGCCTCGGCCCAACAAAATGGCGGCGGC
AGCGGTGTCGCTTTGTTTCCGCGGCTC

horse
GTTTTGTCAATAAGTCCAAGAAAAGAAGGCGGGTCAAAAAGCACTTTCCG
AATAAAGTCAAAATACGTCACTAGCACTTTCAGGTCAATGGGACTGAACT
GGACCCCCCACGACAAGGTAGACGGGATCAAATGACGTAGACCGCCTTA
GCAACAGGCCCCCAAAGAAGGTGAATCTCCGCCGCCAATAAGCGCCTGCG
CCTCGGCGGAGGGAGCCAATGGACGACGGGAAGCGGAAGAGGGAGGCCG
CGCCAATTCTCCTGCCTGAGCCTCGGCCCAAGAAAATGGCGGCGGCGGCG
GCGTCGCTTTTGTTTCCGCGGCTC

dog
GTTTCGTCATTATGTCAAAGAAAAGCGAATGGATCAAAAAGCATTTTCAG
AATAAAGTCAAAATACGTCACTAGCACTTTCAGGTCATTGGGATTGAATC
GGAGCCCCTATGAAAAGGTGGAGGGGATCAAATGACGTAGACCGCCTTA
GCAACAGGCCCCCAAAGAAGGTGAATTTCAGTAGCCAATAAGAGTCTGA
GCTGTGGAAGAGGTAGCCAATGGGTAACGGAAAGAAGAAGAGGGAGGAC
GCGCCAATTCTTCTGCCTGAGCCTCGGCCCAACAAAATGGCGGCGGCGGC
AGCGTCGCTTTGTTTCCGCGGCTC
